# Supplementary material for: Alterations in Whey Protein Abundance Correlated with the Somatic Cell Count Identified via Label-Free and Selected Reaction Monitoring Proteomic Approaches
Source: Animals (Basel). 2025 Feb 26;15(5):675. doi: 10.3390/ani15050675 (PMC11898894; doi:10.3390/ani15050675)
Supplement: Supplementary file 1 [file animals-15-00675-s001.zip › animals-3449227-supplementary.pdf]

Table S1. Milk yield and the contents of protein and fat from the samples with the different somatic cell counts.

Milk composition	S1 group	S2 group	S3 group	S4 group	S5 group
Protein (%)	3.10±0.15b	3.23±0.17ab	3.20±0.23ab	3.24±0.18ab	3.39±0.22a
Fat (%)	3.50±0.35	3.40±0.33	3.42±0.49	3.72±0.28	3.86±0.36
Milk yield （kg）	27.98±5.52	28.43±4.85	27.47±4.23	25.13±4.59	24.73±5.80
Somatic cell count ($\times 10^4$ cells/mL) *	7.2 (6-9)	18.4 (17-20)	40.4 (38-42)	73.6 (68-80)	208.9 (176-243)
Days in milk （day）	175.6 (165-185)	183.6 (177-210)	182.2 (162-207)	199.4 (176-232)	197.1 (152-238)

* Somatic cell count presented with mean and range.

Table S2. The information of the differentially abundant proteins among the five milk groups with the different somatic cell counts.

Accession No.	Protein Name	Gene Name	S1 group	S2 group	S3 group	S4 group	S5 group	S1/S2	S1/S3	S1/S4	S1/S5	-Log p value
P80457	Xanthine dehydrogenase/oxidase	XDH	1.32E+10	1.20E+10	1.10E+10	1.24E+10	8.62E+09	1.10	1.20	1.06	1.53	3.35
P48616	Vimentin	VIM	7.74E+08	8.34E+07	9.14E+07	7.78E+07	8.79E+07	9.28	8.47	9.95	8.81	1.44
G3X7S7	Tropomyosin alpha-1 chain	TPM1	1.29E+09	5.85E+08	0.00E+00	1.69E+06	9.72E+06	2.21	#DIV/0!	765.86	133.14	1.67
F1MN93	DNA topoisomerase I	TOP1	2.88E+07	1.39E+07	0.00E+00	0.00E+00	0.00E+00	2.07	#DIV/0!	#DIV/0!	#DIV/0!	1.96
A6QPW7	TNF receptor superfamily member 6b	TNFRSF6B	1.70E+07	2.28E+07	3.27E+07	2.29E+07	2.21E+07	0.74	0.52	0.74	0.77	1.98
A7E3W4	Transketolase	TKT	1.65E+07	1.86E+07	2.86E+07	3.27E+07	6.96E+07	0.89	0.58	0.50	0.24	4.97
Q29443	Serotransferrin	TF	2.74E+09	4.65E+09	4.95E+09	4.02E+09	4.32E+09	0.59	0.55	0.68	0.64	1.87
A7E355	SYNCRIP protein (Fragment)	SYNCRIP	7.55E+06	3.61E+06	0.00E+00	0.00E+00	3.56E+06	2.09	#DIV/0!	#DIV/0!	2.12	1.50
Q27960	Sodium-dependent phosphate transport protein 2B	SLC34A2	8.44E+08	6.63E+08	8.10E+08	6.79E+08	5.19E+08	1.27	1.04	1.24	1.62	3.04
Q2TBR3	SET nuclear oncogene	SET	4.17E+07	1.18E+07	1.00E+06	0.00E+00	5.52E+05	3.54	41.62	#DIV/0!	75.47	3.23
P50448	Factor XIIa inhibitor	SERPING1	5.26E+08	6.04E+08	4.30E+08	6.05E+08	5.07E+08	0.87	1.22	0.87	1.04	1.69
F1MSZ6	Antithrombin-III	SERPINC1	2.99E+08	4.16E+08	3.76E+08	3.46E+08	2.74E+08	0.72	0.79	0.86	1.09	2.49
A6QPZ4	SERPINB4 protein	SERPINB4	0.00E+00	3.26E+07	6.90E+07	7.72E+07	2.09E+08	0.00	0.00	0.00	0.00	6.15
Q1JPB0	Leukocyte elastase inhibitor	SERPINB1	4.88E+07	9.06E+07	1.07E+08	1.29E+08	2.09E+08	0.54	0.46	0.38	0.23	4.54
A2I7N3	Endopin 2B	SERPINA3-7	3.60E+08	5.31E+08	4.55E+08	5.42E+08	6.48E+08	0.68	0.79	0.66	0.55	2.70
A2I7N1	Serpin A3-5	SERPINA3-5	0.00E+00	0.00E+00	3.88E+06	0.00E+00	1.31E+07	#DIV/0!	0.00	#DIV/0!	0.00	1.34
A2I7M9	Serpin A3-2	SERPINA3-2	5.94E+08	9.62E+08	9.15E+08	1.00E+09	1.29E+09	0.62	0.65	0.59	0.46	4.27
G8JKW7	Uncharacterized protein	SERPINA3	1.71E+08	4.00E+08	3.39E+08	4.10E+08	5.54E+08	0.43	0.50	0.42	0.31	3.01
P34955	Alpha-1-antiproteinase	SERPINA1	3.03E+08	3.85E+08	3.71E+08	3.86E+08	3.54E+08	0.79	0.82	0.78	0.85	1.64
Q0VCW4	L-serine dehydratase/L-threonine	SDS	0.00E+00	0.00E+00	4.09E+06	3.58E+06	2.40E+07	#DIV/0!	0.00	0.00	0.00	1.86

	deaminase											
Q8SQ28	Serum amyloid A protein	SAA3	2.81E+07	5.25E+07	1.06E+08	1.42E+08	1.60E+08	0.53	0.27	0.20	0.18	5.59
P61284	60S ribosomal protein L12	RPL12	3.44E+07	1.23E+07	0.00E+00	0.00E+00	0.00E+00	2.79	#DIV/0!	#DIV/0!	#DIV/0!	1.77
Q58DP6	Ribonuclease 4	RNASE4	1.81E+09	1.86E+09	2.09E+09	2.32E+09	1.61E+09	0.98	0.87	0.78	1.13	2.64
Q762I5	Resistin	RETN	0.00E+00	1.06E+07	2.01E+07	4.64E+07	9.86E+07	0.00	0.00	0.00	0.00	4.71
P18902	Retinol-binding protein 4	RBP4	2.21E+07	2.50E+07	2.21E+07	2.19E+07	1.63E+07	0.88	1.00	1.01	1.35	1.31
A1L528	RAB1A, member RAS oncogene family	RAB1A	1.02E+08	5.37E+07	5.97E+07	6.09E+07	5.39E+07	1.90	1.71	1.68	1.90	1.41
P58073	Parathyroid hormone-related protein	PTHLH	3.17E+07	5.29E+07	8.86E+06	8.36E+06	5.44E+06	0.60	3.58	3.80	5.83	1.49
O02853	Prostaglandin-H2 D-isomerase	PTGDS	6.27E+08	1.48E+09	1.09E+09	1.51E+09	1.94E+09	0.42	0.57	0.41	0.32	2.68
A1L555	Prosaposin	PSAP	2.60E+08	3.59E+08	3.40E+08	2.86E+08	2.06E+08	0.72	0.76	0.91	1.26	2.07
P80311	Peptidyl-prolyl cis-trans isomerase B	PPIB	8.69E+07	8.78E+07	6.27E+07	7.12E+07	4.57E+07	0.99	1.39	1.22	1.90	2.09
P62935	Peptidyl-prolyl cis-trans isomerase A	PPIA	1.55E+08	1.20E+08	9.45E+07	8.16E+07	8.42E+07	1.29	1.64	1.89	1.84	1.95
P06868	Plasminogen	PLG	1.43E+08	2.09E+08	2.30E+08	2.21E+08	2.19E+08	0.69	0.62	0.65	0.66	1.55
Q8SPP7	Peptidoglycan recognition protein 1	PGLYRP1	7.56E+07	4.36E+08	7.68E+08	1.14E+09	2.57E+09	0.17	0.10	0.07	0.03	10.97
Q0V8C4	Protein disulfide isomerase-associated 6 (Fragment)	PDIA6	4.94E+08	0.00E+00	6.54E+06	2.41E+07	2.22E+07	#DIV/0!	75.57	20.47	22.21	3.66
P38657	Protein disulfide-isomerase A3	PDIA3	1.70E+07	4.40E+06	1.99E+06	0.00E+00	7.81E+06	3.85	8.50	#DIV/0!	2.17	2.34
Q3SYT9	Poly(RC) binding protein 2	PCBP2	2.01E+07	1.06E+07	0.00E+00	0.00E+00	3.92E+06	1.90	#DIV/0!	#DIV/0!	5.14	1.89
p02754	Major allergen beta-lactoglobulin	PAEP	3.28E+11	3.69E+11	3.57E+11	3.15E+11	2.73E+11	0.89	0.92	1.04	1.20	3.77
Q3ZBH5	Proliferation-associated 2G4, 38kDa	PA2G4	8.52E+06	8.43E+06	0.00E+00	0.00E+00	1.58E+06	1.01	#DIV/0!	#DIV/0!	5.39	2.18

Q3MHX6	Protein OS-9	OS9	2.01E+07	1.96E+07	2.65E+07	1.80E+07	1.81E+07	1.02	0.76	1.12	1.11	1.53
Q0IIH5	Nucleobindin 2	NUCB2	4.40E+08	4.65E+08	3.95E+08	3.81E+08	2.95E+08	0.95	1.11	1.15	1.49	3.48
Q0P569	Nucleobindin-1	NUCB1	1.03E+09	7.26E+08	4.35E+08	6.44E+08	7.24E+08	1.42	2.38	1.61	1.43	3.00
P79345	NPC intracellular cholesterol transporter 2	NPC2	2.49E+09	3.00E+09	2.58E+09	2.01E+09	1.75E+09	0.83	0.97	1.24	1.42	3.33
Q8WXI7	Uncharacterized protein	MUC16	4.43E+07	4.40E+07	4.08E+07	6.77E+07	5.66E+07	1.01	1.08	0.65	0.78	1.47
Q56J78	Serum amyloid A protein	M-SAA3.2	1.12E+08	2.56E+08	3.81E+08	4.88E+08	7.27E+08	0.44	0.29	0.23	0.15	6.45
P24627	Lactoferrin (Fragment)	LTF	5.43E+10	8.02E+10	1.03E+11	8.87E+10	9.57E+10	0.68	0.53	0.61	0.57	2.33
P80025	Lactoperoxidase	LPO	8.25E+09	8.52E+09	6.15E+09	6.44E+09	5.42E+09	0.97	1.34	1.28	1.52	2.76
P11151	Lipoprotein lipase	LPL	2.55E+08	1.88E+08	1.65E+08	1.52E+08	1.33E+08	1.36	1.55	1.67	1.91	1.44
A5PJH7	LOC788112 protein	LOC788112	0.00E+00	8.08E+06	2.57E+07	2.56E+07	7.79E+07	0.00	0.00	0.00	0.00	2.98
M5FKI8	Pancreatic adenocarcinoma upregulated factor-like	LOC100295702	2.66E+08	3.25E+08	5.81E+08	4.48E+08	6.43E+08	0.82	0.46	0.59	0.41	3.31
Q3ZC00	Lymphocyte cytosolic protein 1 (L-plastin)	LCP1	3.60E+06	7.97E+06	2.43E+07	2.82E+07	5.71E+07	0.45	0.15	0.13	0.06	2.23
E1B6Z6	Lipocalin 2	LCN2	1.81E+08	3.88E+08	6.62E+08	6.95E+08	1.28E+09	0.47	0.27	0.26	0.14	9.30
Q2TBI0	Lipopolysaccharide-binding protein	LBP	1.76E+08	1.92E+08	2.65E+08	2.72E+08	2.99E+08	0.91	0.66	0.65	0.59	2.20
F1MNT4	Laminin subunit beta 1	LAMB1	1.09E+07	1.14E+07	1.20E+07	6.84E+06	0.00E+00	0.96	0.91	1.59	#DIV/0!	2.21
P01045	Kininogen-2	KNG2	0.00E+00	1.88E+07	2.19E+07	3.70E+07	2.25E+07	0.00	0.00	0.00	0.00	5.57
F1MNV5	Kininogen-1	KNG1	3.94E+08	4.25E+08	4.87E+08	3.95E+08	3.61E+08	0.93	0.81	1.00	1.09	1.33
Q3SYR8	Immunoglobulin J chain	JCHAIN	6.87E+08	7.66E+08	9.16E+08	9.93E+08	1.04E+09	0.90	0.75	0.69	0.66	1.50
Q5EA67	Inter-alpha (Globulin) inhibitor H4 (Plasma Kallikrein-sensitive glycoprotein)	ITIH4	7.14E+07	1.04E+08	7.50E+07	8.32E+07	1.47E+08	0.68	0.95	0.86	0.49	2.23
G3MYN2	Interleukin 18 binding protein	IL18BP	0.00E+00	0.00E+00	0.00E+00	8.36E+06	1.56E+07	#DIV/0!	#DIV/0!	0.00	0.00	3.62

Q05B55	Uncharacterized protein	IGK	2.36E+09	2.33E+09	2.46E+09	3.16E+09	3.31E+09	1.02	0.96	0.75	0.71	2.93
Q9XSG3	Isocitrate dehydrogenase [NADP] cytoplasmic	IDH1	2.10E+08	1.60E+08	1.44E+08	1.73E+08	1.48E+08	1.31	1.46	1.21	1.42	1.44
C6KGD8	Histatherin	HSTN	1.44E+08	1.66E+08	2.05E+08	1.75E+08	1.07E+08	0.86	0.70	0.82	1.35	1.98
P19120	Heat shock cognate 71 kDa protein	HSPA8	1.01E+09	4.77E+08	1.72E+08	1.83E+08	2.73E+08	2.11	5.86	5.50	3.68	1.34
F1MWU9	Heat shock protein family A (Hsp70) member 6	HSPA6	1.45E+08	6.47E+07	2.69E+07	2.68E+07	3.06E+07	2.24	5.38	5.40	4.74	2.24
Q0VCX2	Endoplasmic reticulum chaperone BiP	HSPA5	1.92E+08	9.29E+07	5.93E+07	6.74E+07	9.10E+07	2.06	3.24	2.85	2.11	1.42
P0CB32	Heat shock 70 kDa protein 1-like	HSPA1L	6.70E+06	1.39E+07	3.74E+06	0.00E+00	0.00E+00	0.48	1.79	#DIV/0!	#DIV/0!	1.77
Q3SZV7	Hemopexin	HPX	3.36E+08	4.58E+08	5.94E+08	5.82E+08	7.92E+08	0.73	0.57	0.58	0.42	3.92
G3X6K8	Haptoglobin	HP	1.96E+08	9.43E+07	2.56E+08	4.07E+08	8.11E+08	2.08	0.76	0.48	0.24	1.33
Q3T0D0	Heterogeneous nuclear ribonucleoprotein K	HNRNPK	3.44E+07	1.73E+07	0.00E+00	0.00E+00	3.67E+06	1.99	#DIV/0!	#DIV/0!	9.38	1.32
Q5E9J1	Heterogeneous nuclear ribonucleoprotein F	HNRNPF	4.80E+06	0.00E+00	0.00E+00	0.00E+00	3.21E+06	#DIV/0!	#DIV/0!	#DIV/0!	1.50	1.55
E1BEG2	Heterogeneous nuclear ribonucleoprotein A3	HNRNPA3	1.30E+08	4.22E+07	8.80E+06	3.53E+06	3.58E+06	3.08	14.74	36.70	36.28	1.58
P09867	Heterogeneous nuclear ribonucleoprotein A1	HNRNPA1	1.72E+08	1.03E+08	1.98E+06	3.08E+06	3.13E+06	1.67	86.98	55.97	55.13	1.50
Q3SX14	Gelsolin	GSN	3.08E+08	2.61E+08	3.15E+08	2.81E+08	2.02E+08	1.18	0.98	1.10	1.52	3.08
Q2HJI6	Granulin	GRN	2.27E+08	2.94E+08	3.30E+08	2.68E+08	1.74E+08	0.77	0.69	0.85	1.31	2.48
P80195	Glycosylation-dependent cell adhesion molecule 1	GLYCAM1	1.44E+10	1.95E+10	1.38E+10	1.61E+10	8.95E+09	0.74	1.04	0.89	1.61	1.83
Q3MHN5	Vitamin D binding protein	GC	1.01E+09	1.09E+09	1.12E+09	9.32E+08	7.02E+08	0.93	0.90	1.08	1.44	2.55

Q3SZZ9	FGG protein	FGG	3.37E+08	4.76E+08	4.71E+08	4.81E+08	6.35E+08	0.71	0.71	0.70	0.53	2.35
Q9MZ06	Fibroblast growth factor-binding protein 1	FGFBP1	1.04E+09	1.31E+09	1.25E+09	1.10E+09	9.19E+08	0.80	0.84	0.95	1.13	1.89
F1MAV0	Fibrinogen beta chain	FGB	1.80E+08	3.29E+08	3.77E+08	3.88E+08	5.92E+08	0.55	0.48	0.46	0.30	3.75
P02672	Fibrinogen alpha chain	FGA	1.73E+08	2.56E+08	3.31E+08	3.23E+08	4.92E+08	0.68	0.52	0.54	0.35	2.74
P10790	Fatty acid-binding protein, heart	FABP3	4.66E+09	5.33E+09	4.50E+09	4.25E+09	2.89E+09	0.88	1.04	1.10	1.61	2.73
P15396	Ectonucleotide pyrophosphatase/phosphodiesterase family member 3	ENPP3	6.11E+07	3.95E+07	4.02E+07	4.12E+07	3.67E+07	1.55	1.52	1.48	1.67	1.65
Q3SZ54	Eukaryotic initiation factor 4A-I	EIF4A1	4.81E+07	1.03E+07	0.00E+00	0.00E+00	9.80E+06	4.66	#DIV/0!	#DIV/0!	4.91	1.67
Q3ZC64	Ephrin-A1	EFNA1	9.51E+06	9.64E+06	1.47E+07	1.13E+07	1.42E+07	0.99	0.65	0.84	0.67	1.30
O62654	Desmin	DES	8.37E+07	2.73E+07	0.00E+00	0.00E+00	0.00E+00	3.07	#DIV/0!	#DIV/0!	#DIV/0!	1.96
P46160	Beta-defensin 2	DEFB2	0.00E+00	1.07E+07	2.27E+07	2.06E+07	9.07E+07	0.00	0.00	0.00	0.00	5.59
P46171	Beta-defensin 12	DEFB12	0.00E+00	1.24E+07	2.08E+07	2.97E+07	9.25E+07	0.00	0.00	0.00	0.00	6.58
P07688	Cathepsin B	CTSB	1.03E+08	1.48E+08	1.73E+08	1.75E+08	1.49E+08	0.70	0.60	0.59	0.70	2.60
Q5DPW9	Cystatin	CST6	1.37E+08	1.61E+08	1.66E+08	1.70E+08	1.11E+08	0.85	0.83	0.81	1.24	2.47
P02668	Kappa casein (Fragment)	CSN3	1.72E+10	1.83E+10	2.07E+10	1.77E+10	1.43E+10	0.94	0.83	0.97	1.20	1.57
P02662	Alpha-S1-casein	CSN1S1	2.35E+10	3.50E+10	4.86E+10	5.09E+10	4.47E+10	0.67	0.48	0.46	0.52	2.42
F1MFI4	Cartilage acidic protein 1	CRTAC1	2.64E+07	2.53E+07	3.85E+07	2.48E+07	2.18E+07	1.04	0.68	1.06	1.21	1.33
F6R3I5	Cysteine-rich secretory protein 3 precursor	CRISP3	2.01E+09	2.62E+09	2.85E+09	2.43E+09	2.29E+09	0.77	0.71	0.83	0.88	1.42
F1MX50	Cellular repressor of E1A stimulated genes 1	CREG1	9.25E+08	1.02E+09	8.35E+08	1.35E+09	7.12E+08	0.90	1.11	0.69	1.30	1.64
F1N076	Ceruloplasmin	CP	8.07E+07	9.89E+07	1.23E+08	1.22E+08	1.45E+08	0.82	0.66	0.66	0.56	3.40
P17697	Clusterin	CLU	4.48E+08	3.60E+08	6.14E+08	6.42E+08	7.85E+08	1.24	0.73	0.70	0.57	2.32
Q2KIS7	Tetranectin	CLEC3B	2.20E+08	2.51E+08	2.15E+08	1.71E+08	1.41E+08	0.88	1.02	1.29	1.56	2.59

G3X7D2	Chitinase-3-like protein 1	CHI3L1	2.06E+08	2.51E+08	4.28E+08	4.29E+08	5.45E+08	0.82	0.48	0.48	0.38	1.70
Q28085	Complement factor H	CFH	6.67E+07	6.32E+07	8.92E+07	9.74E+07	1.01E+08	1.06	0.75	0.69	0.66	1.49
P81187	Complement factor B	CFB	2.09E+08	2.75E+08	2.90E+08	3.04E+08	3.16E+08	0.76	0.72	0.69	0.66	2.31
F1N619	Cadherin-1	CDH1	1.02E+08	1.25E+08	1.40E+08	9.61E+07	7.52E+07	0.81	0.73	1.06	1.36	3.07
A6QNW7	CD5L protein	CD5L	5.89E+08	6.27E+08	8.77E+08	7.98E+08	6.61E+08	0.94	0.67	0.74	0.89	1.48
P26201	Platelet glycoprotein 4	CD36	2.09E+09	1.71E+09	1.75E+09	1.83E+09	1.38E+09	1.22	1.19	1.14	1.52	1.79
Q95122	Monocyte differentiation antigen CD14	CD14	6.45E+08	5.88E+08	4.70E+08	5.36E+08	4.63E+08	1.10	1.37	1.20	1.39	2.52
Q3ZCI9	T-complex protein 1 subunit theta	CCT8	2.92E+06	2.44E+06	0.00E+00	0.00E+00	5.18E+05	1.20	#DIV/0!	#DIV/0!	5.64	2.16
P56425	Cathelicidin-7	CATHL7	0.00E+00	1.73E+07	2.40E+07	3.02E+07	6.20E+07	0.00	0.00	0.00	0.00	2.82
P54228	Cathelicidin-6	CATHL6	0.00E+00	9.04E+06	2.67E+07	3.80E+07	9.12E+07	0.00	0.00	0.00	0.00	8.03
P54229	Cathelicidin-5	CATHL5	0.00E+00	5.27E+07	9.65E+07	1.65E+08	3.67E+08	0.00	0.00	0.00	0.00	12.42
P33046	Cathelicidin-4	CATHL4	1.49E+07	1.20E+08	2.95E+08	4.20E+08	1.11E+09	0.12	0.05	0.04	0.01	7.69
P19661	Cathelicidin-3	CATHL3	4.59E+06	6.77E+07	1.03E+08	2.01E+08	4.83E+08	0.07	0.04	0.02	0.01	7.42
P19660	Cathelicidin-2	CATHL2	3.72E+07	2.33E+08	4.08E+08	4.70E+08	1.12E+09	0.16	0.09	0.08	0.03	8.33
P22226	Cathelicidin-1	CATHL1	2.33E+07	3.49E+08	7.76E+08	9.70E+08	1.90E+09	0.07	0.03	0.02	0.01	6.64
P80209	Cathepsin D (Fragment)	cat-D	1.27E+07	1.52E+07	1.97E+07	1.64E+07	1.38E+07	0.84	0.65	0.77	0.92	1.33
E1BH06	Uncharacterized protein	C4A	1.23E+08	1.56E+08	1.67E+08	1.65E+08	1.71E+08	0.78	0.73	0.74	0.72	1.62
Q2UVX4	Complement C3	C3	6.25E+09	7.49E+09	9.09E+09	8.80E+09	8.99E+09	0.83	0.69	0.71	0.70	1.54
P18892	Butyrophilin subfamily 1 member A1	BTN1A1	3.91E+09	2.83E+09	2.97E+09	3.03E+09	2.41E+09	1.38	1.32	1.29	1.62	2.21
P01888	Beta-2-microglobulin (Fragment)	B2M	2.66E+09	2.85E+09	2.49E+09	2.35E+09	1.87E+09	0.93	1.07	1.14	1.42	2.46
G3N0Q8	Azurocidin 1	AZU1	0.00E+00	2.58E+06	6.40E+07	1.02E+08	2.62E+08	0.00	0.00	0.00	0.00	5.85
P00829	ATP synthase subunit beta, mitochondrial	ATP5F1B	5.59E+06	1.61E+07	0.00E+00	0.00E+00	0.00E+00	0.35	#DIV/0!	#DIV/0!	#DIV/0!	1.31
A2VDL6	Sodium/potassium-transporting	ATP1A2	1.37E+07	4.97E+06	0.00E+00	0.00E+00	1.22E+06	2.75	#DIV/0!	#DIV/0!	11.24	1.44

	ATPase subunit alpha-2													
Q9TU03	Rho GDP-dissociation inhibitor 2	ARHGDIB	0.00E+00	0.00E+00	1.66E+07	2.72E+07	4.50E+07	#DIV/0!	0.00	0.00	0.00	4.14
P17690	Beta-2-glycoprotein 1	APOH	3.80E+08	4.09E+08	4.17E+08	3.41E+08	3.25E+08	0.93	0.91	1.12	1.17	1.32
P00711	Alpha lactalbumin (Fragment)	LALBA	1.01E+11	1.14E+11	1.02E+11	9.56E+10	7.21E+10	0.89	0.99	1.06	1.40	3.67
A6QLL8	Fructose-bisphosphate aldolase	ALDOA	1.00E+09	4.94E+08	1.63E+08	1.83E+08	2.72E+08	2.03	6.15	5.49	3.69	1.91
A0A140T897	Serum albumin	ALB	1.13E+11	1.33E+11	1.28E+11	1.15E+11	9.62E+10	0.85	0.89	0.98	1.18	2.24
Q9TUM6	Perilipin	ADRP	6.13E+08	5.20E+08	4.53E+08	5.37E+08	4.35E+08	1.18	1.35	1.14	1.41	2.32
P63258	Actin, cytoplasmic 2	ACTG1	4.29E+09	1.37E+09	7.48E+08	9.73E+08	1.63E+09	3.13	5.74	4.41	2.64	1.87
Q4GZT4	ATP-binding cassette sub-family G member 2	ABCG2	3.47E+08	2.40E+08	2.91E+08	3.16E+08	2.23E+08	1.45	1.19	1.10	1.55	2.23
Q7SIH1	Alpha-2-macroglobulin	A2M	3.56E+08	4.63E+08	5.83E+08	5.57E+08	7.51E+08	0.77	0.61	0.64	0.47	2.02
Q2KJF1	Alpha-1B-glycoprotein	A1BG	3.39E+08	5.96E+08	5.67E+08	4.79E+08	5.09E+08	0.57	0.60	0.71	0.67	3.94
G3N0V0	Uncharacterized protein		1.99E+09	2.53E+09	3.57E+09	3.73E+09	6.12E+09	0.78	0.56	0.53	0.32	5.56
Q28133	Allergen Bos d 2		0.00E+00	1.31E+07	5.88E+07	3.39E+07	8.88E+06	0.00	0.00	0.00	0.00	1.89
G3N342	Uncharacterized protein		0.00E+00	8.93E+06	2.08E+07	4.32E+07	2.51E+07	0.00	0.00	0.00	0.00	1.44
A0A0A0MPA0	Uncharacterized protein		4.09E+07	1.08E+08	4.34E+07	1.95E+08	2.43E+08	0.38	0.94	0.21	0.17	1.31
A5D7Q2	Uncharacterized protein		1.59E+09	1.71E+09	1.81E+09	2.45E+09	3.03E+09	0.93	0.88	0.65	0.52	1.60
G5E513	Uncharacterized protein		6.44E+09	8.77E+09	1.03E+10	9.40E+09	8.78E+09	0.73	0.63	0.69	0.73	3.40
Q2KIT0	Protein HP-20 homolog		7.59E+07	9.11E+07	1.11E+08	1.02E+08	9.72E+07	0.83	0.68	0.75	0.78	1.79

Table S3. The protein-protein interactions of differentially abundant proteins in milk with different somatic cell count predicted by STRING software

Node1	Node2	Node1_string_id	Node2_string_id	Experimentally determined interaction	Database annotated	Automated textmining	Combined score
A1BG	CRISP3	9913.ENSBTAP00000069446	9913.ENSBTAP00000017167	0.077	0	0.837	0.843
A2M	PLG	9913.ENSBTAP00000006167	9913.ENSBTAP00000001674	0.141	0	0.687	0.719
A2M	APOH	9913.ENSBTAP00000006167	9913.ENSBTAP00000002492	0.075	0	0.705	0.716
A2M	CP	9913.ENSBTAP00000006167	9913.ENSBTAP00000016137	0	0	0.798	0.798
A2M	TF	9913.ENSBTAP00000006167	9913.ENSBTAP00000009564	0	0	0.795	0.795
A2M	ALB	9913.ENSBTAP00000006167	9913.ENSBTAP00000022763	0.064	0	0.803	0.808
A2M	HSPA5	9913.ENSBTAP00000006167	9913.ENSBTAP00000052422	0.083	0	0.808	0.817
A2M	SERPINA1	9913.ENSBTAP00000006167	9913.ENSBTAP00000065816	0	0	0.862	0.862
A2M	HP	9913.ENSBTAP00000006167	9913.ENSBTAP00000071329	0.133	0	0.856	0.869
ALB	PLG	9913.ENSBTAP00000022763	9913.ENSBTAP00000001674	0.047	0	0.855	0.855
ALB	LTF	9913.ENSBTAP00000022763	9913.ENSBTAP00000001704	0	0	0.995	0.995
ALB	HPX	9913.ENSBTAP00000022763	9913.ENSBTAP00000004635	0.102	0.5	0.699	0.853
ALB	A2M	9913.ENSBTAP00000022763	9913.ENSBTAP00000006167	0.064	0	0.803	0.808
ALB	CLU	9913.ENSBTAP00000022763	9913.ENSBTAP00000007324	0	0	0.768	0.768
ALB	TF	9913.ENSBTAP00000022763	9913.ENSBTAP00000009564	0	0	0.835	0.835
ALB	CP	9913.ENSBTAP00000022763	9913.ENSBTAP00000016137	0	0	0.808	0.808
ALB	B2M	9913.ENSBTAP00000022763	9913.ENSBTAP00000016359	0.262	0	0.811	0.854
ALB	CD36	9913.ENSBTAP00000022763	9913.ENSBTAP00000066341	0	0	0.745	0.745
ALB	HSPA5	9913.ENSBTAP00000022763	9913.ENSBTAP00000052422	0	0	0.741	0.741
ALB	CSN3	9913.ENSBTAP00000022763	9913.ENSBTAP00000028685	0	0	0.797	0.797
ALB	LPO	9913.ENSBTAP00000022763	9913.ENSBTAP00000054415	0	0	0.796	0.796
ALB	C3	9913.ENSBTAP00000022763	9913.ENSBTAP00000022979	0.077	0	0.798	0.806
ALB	FGB	9913.ENSBTAP00000022763	9913.ENSBTAP00000067912	0.041	0	0.72	0.72

ALB	KNG1	9913.ENSBTAP00000022763	9913.ENSBTAP00000048995	0	0	0.835	0.835
ALB	CDH1	9913.ENSBTAP00000022763	9913.ENSBTAP00000073179	0	0	0.878	0.878
ALB	SERPINA1	9913.ENSBTAP00000022763	9913.ENSBTAP00000065816	0.063	0	0.883	0.885
ALB	HP	9913.ENSBTAP00000022763	9913.ENSBTAP00000071329	0.067	0	0.886	0.889
ALB	LALBA	9913.ENSBTAP00000022763	9913.ENSBTAP00000060771	0	0	0.997	0.997
ALB	PAEP	9913.ENSBTAP00000022763	9913.ENSBTAP00000062602	0	0	0.998	0.998
ALDOA	TKT	9913.ENSBTAP00000057869	9913.ENSBTAP00000004892	0.077	0.939	0.631	0.977
APOH	A2M	9913.ENSBTAP00000002492	9913.ENSBTAP00000006167	0.075	0	0.705	0.716
APOH	LPL	9913.ENSBTAP00000002492	9913.ENSBTAP00000017086	0.043	0.9	0.29	0.926
B2M	ALB	9913.ENSBTAP00000016359	9913.ENSBTAP00000022763	0.262	0	0.811	0.854
B2M	PDIA3	9913.ENSBTAP00000016359	9913.ENSBTAP00000022854	0.276	0.5	0.872	0.949
BTN1A1	PLIN2	9913.ENSBTAP00000042450	9913.ENSBTAP00000007519	0.063	0	0.735	0.741
BTN1A1	XDH	9913.ENSBTAP00000042450	9913.ENSBTAP00000061483	0	0.9	0.861	0.985
C3	PLG	9913.ENSBTAP00000022979	9913.ENSBTAP00000001674	0.141	0.9	0.711	0.973
C3	C4A	9913.ENSBTAP00000022979	9913.ENSBTAP00000009019	0	0.9	0.341	0.931
C3	TF	9913.ENSBTAP00000022979	9913.ENSBTAP00000009564	0.044	0	0.748	0.748
C3	CFB	9913.ENSBTAP00000022979	9913.ENSBTAP00000009800	0.311	0.9	0.668	0.975
C3	ALB	9913.ENSBTAP00000022979	9913.ENSBTAP00000022763	0.077	0	0.798	0.806
C3	KNG1	9913.ENSBTAP00000022979	9913.ENSBTAP00000048995	0	0.4	0.585	0.74
C3	SERPINA1	9913.ENSBTAP00000022979	9913.ENSBTAP00000065816	0	0	0.799	0.799
C3	HP	9913.ENSBTAP00000022979	9913.ENSBTAP00000071329	0.133	0	0.741	0.765
C3	CFH	9913.ENSBTAP00000022979	9913.ENSBTAP00000073132	0.463	0.9	0.35	0.962
C4A	CFB	9913.ENSBTAP00000009019	9913.ENSBTAP00000009800	0.311	0.5	0.666	0.875
C4A	C3	9913.ENSBTAP00000009019	9913.ENSBTAP00000022979	0	0.9	0.341	0.931
CD14	LBP	9913.ENSBTAP00000020009	9913.ENSBTAP00000022428	0.187	0.6	0.843	0.944
CD36	PLIN2	9913.ENSBTAP00000066341	9913.ENSBTAP00000007519	0.112	0	0.697	0.719

CD36	LPL	9913.ENSBTAP00000066341	9913.ENSBTAP00000017086	0	0	0.758	0.758
CD36	ALB	9913.ENSBTAP00000066341	9913.ENSBTAP00000022763	0	0	0.745	0.745
CDH1	ALB	9913.ENSBTAP00000073179	9913.ENSBTAP00000022763	0	0	0.878	0.878
CDH1	VIM	9913.ENSBTAP00000073179	9913.ENSBTAP00000024572	0.043	0	0.865	0.865
CFB	C4A	9913.ENSBTAP00000009800	9913.ENSBTAP00000009019	0.311	0.5	0.666	0.875
CFB	SERPINA1	9913.ENSBTAP00000009800	9913.ENSBTAP00000065816	0.071	0	0.707	0.716
CFB	CFH	9913.ENSBTAP00000009800	9913.ENSBTAP00000073132	0.105	0.9	0.405	0.942
CFB	C3	9913.ENSBTAP00000009800	9913.ENSBTAP00000022979	0.311	0.9	0.668	0.975
CFH	CFB	9913.ENSBTAP00000073132	9913.ENSBTAP00000009800	0.105	0.9	0.405	0.942
CFH	C3	9913.ENSBTAP00000073132	9913.ENSBTAP00000022979	0.463	0.9	0.35	0.962
CLEC3B	PLG	9913.ENSBTAP00000024391	9913.ENSBTAP00000001674	0.063	0	0.835	0.839
CLU	LTF	9913.ENSBTAP00000007324	9913.ENSBTAP00000001704	0.075	0.5	0.708	0.853
CLU	HP	9913.ENSBTAP00000007324	9913.ENSBTAP00000071329	0.078	0	0.76	0.769
CLU	ALB	9913.ENSBTAP00000007324	9913.ENSBTAP00000022763	0	0	0.768	0.768
CP	HPX	9913.ENSBTAP00000016137	9913.ENSBTAP00000004635	0.077	0	0.709	0.72
CP	A2M	9913.ENSBTAP00000016137	9913.ENSBTAP00000006167	0	0	0.798	0.798
CP	TF	9913.ENSBTAP00000016137	9913.ENSBTAP00000009564	0.078	0	0.77	0.778
CP	ALB	9913.ENSBTAP00000016137	9913.ENSBTAP00000022763	0	0	0.808	0.808
CP	SERPINA1	9913.ENSBTAP00000016137	9913.ENSBTAP00000065816	0.077	0	0.842	0.847
CP	HP	9913.ENSBTAP00000016137	9913.ENSBTAP00000071329	0.084	0	0.885	0.89
CRISP3	A1BG	9913.ENSBTAP00000017167	9913.ENSBTAP00000069446	0.077	0	0.837	0.843
CSN1S1	LTF	9913.ENSBTAP00000010119	9913.ENSBTAP00000001704	0	0	0.777	0.777
CSN1S1	PAEP	9913.ENSBTAP00000010119	9913.ENSBTAP00000062602	0	0	0.889	0.889
CSN1S1	LALBA	9913.ENSBTAP00000010119	9913.ENSBTAP00000060771	0	0	0.936	0.936
CSN1S1	CSN3	9913.ENSBTAP00000010119	9913.ENSBTAP00000028685	0.047	0	0.984	0.984
CSN3	PLG	9913.ENSBTAP00000028685	9913.ENSBTAP00000001674	0.048	0	0.722	0.724

CSN3	LTF	9913.ENSBTAP00000028685	9913.ENSBTAP00000001704	0	0	0.825	0.825
CSN3	CSN1S1	9913.ENSBTAP00000028685	9913.ENSBTAP00000010119	0.047	0	0.984	0.984
CSN3	GLYCAM1	9913.ENSBTAP00000028685	9913.ENSBTAP00000017854	0	0	0.729	0.729
CSN3	ALB	9913.ENSBTAP00000028685	9913.ENSBTAP00000022763	0	0	0.797	0.797
CSN3	LPO	9913.ENSBTAP00000028685	9913.ENSBTAP00000054415	0	0	0.724	0.724
CSN3	LALBA	9913.ENSBTAP00000028685	9913.ENSBTAP00000060771	0	0	0.951	0.951
CSN3	PAEP	9913.ENSBTAP00000028685	9913.ENSBTAP00000062602	0.045	0	0.997	0.997
DES	VIM	9913.ENSBTAP00000007041	9913.ENSBTAP00000024572	0	0.8	0	0.8
FGA	SERPINC1	9913.ENSBTAP00000002145	9913.ENSBTAP00000072452	0.043	0	0.707	0.708
FGA	FGB	9913.ENSBTAP00000002145	9913.ENSBTAP00000067912	0.992	0.9	0.689	0.999
FGA	FGG	9913.ENSBTAP00000002145	9913.ENSBTAP00000008877	0.992	0.9	0.685	0.999
FGB	FGA	9913.ENSBTAP00000067912	9913.ENSBTAP00000002145	0.992	0.9	0.689	0.999
FGB	FGG	9913.ENSBTAP00000067912	9913.ENSBTAP00000008877	0.983	0.9	0.688	0.999
FGB	ALB	9913.ENSBTAP00000067912	9913.ENSBTAP00000022763	0.041	0	0.72	0.72
FGB	KNG1	9913.ENSBTAP00000067912	9913.ENSBTAP00000048995	0.09	0	0.741	0.754
FGG	FGA	9913.ENSBTAP00000008877	9913.ENSBTAP00000002145	0.992	0.9	0.685	0.999
FGG	FGB	9913.ENSBTAP00000008877	9913.ENSBTAP00000067912	0.983	0.9	0.688	0.999
GC	TF	9913.ENSBTAP00000062210	9913.ENSBTAP00000009564	0.067	0	0.799	0.804
GC	RBP4	9913.ENSBTAP00000062210	9913.ENSBTAP00000057660	0	0	0.747	0.747
GC	SERPINA1	9913.ENSBTAP00000062210	9913.ENSBTAP00000065816	0.077	0	0.689	0.701
GC	HP	9913.ENSBTAP00000062210	9913.ENSBTAP00000071329	0.072	0	0.732	0.741
GLYCAM1	CSN3	9913.ENSBTAP00000017854	9913.ENSBTAP00000028685	0	0	0.729	0.729
HNRNPA1	HNRNPF	9913.ENSBTAP00000013110	9913.ENSBTAP00000011658	0.167	0.4	0.751	0.865
HNRNPA1	PCBP2	9913.ENSBTAP00000013110	9913.ENSBTAP00000065293	0.134	0.4	0.786	0.879
HNRNPA1	HNRNPK	9913.ENSBTAP00000013110	9913.ENSBTAP00000028162	0.233	0.4	0.796	0.897
HNRNPA3	HNRNPF	9913.ENSBTAP00000028757	9913.ENSBTAP00000011658	0.167	0.4	0.58	0.772

HNRNPA3	HNRNPK	9913.ENSBTAP00000028757	9913.ENSBTAP00000028162	0.233	0.4	0.504	0.751
HNRNPA3	PCBP2	9913.ENSBTAP00000028757	9913.ENSBTAP00000065293	0.134	0.4	0.493	0.713
HNRNPF	HNRNPA3	9913.ENSBTAP00000011658	9913.ENSBTAP00000028757	0.167	0.4	0.58	0.772
HNRNPF	HNRNPA1	9913.ENSBTAP00000011658	9913.ENSBTAP00000013110	0.167	0.4	0.751	0.865
HNRNPF	HNRNPK	9913.ENSBTAP00000011658	9913.ENSBTAP00000028162	0.142	0.4	0.785	0.879
HNRNPK	HNRNPF	9913.ENSBTAP00000028162	9913.ENSBTAP00000011658	0.142	0.4	0.785	0.879
HNRNPK	HNRNPA1	9913.ENSBTAP00000028162	9913.ENSBTAP00000013110	0.233	0.4	0.796	0.897
HNRNPK	HNRNPA3	9913.ENSBTAP00000028162	9913.ENSBTAP00000028757	0.233	0.4	0.504	0.751
HP	LTF	9913.ENSBTAP00000071329	9913.ENSBTAP00000001704	0.064	0	0.788	0.793
HP	HPX	9913.ENSBTAP00000071329	9913.ENSBTAP00000004635	0.073	0	0.918	0.921
HP	A2M	9913.ENSBTAP00000071329	9913.ENSBTAP00000006167	0.133	0	0.856	0.869
HP	CLU	9913.ENSBTAP00000071329	9913.ENSBTAP00000007324	0.078	0	0.76	0.769
HP	TF	9913.ENSBTAP00000071329	9913.ENSBTAP00000009564	0.064	0	0.744	0.75
HP	CP	9913.ENSBTAP00000071329	9913.ENSBTAP00000016137	0.084	0	0.885	0.89
HP	LBP	9913.ENSBTAP00000071329	9913.ENSBTAP00000022428	0	0	0.784	0.784
HP	ALB	9913.ENSBTAP00000071329	9913.ENSBTAP00000022763	0.067	0	0.886	0.889
HP	C3	9913.ENSBTAP00000071329	9913.ENSBTAP00000022979	0.133	0	0.741	0.765
HP	GC	9913.ENSBTAP00000071329	9913.ENSBTAP00000062210	0.072	0	0.732	0.741
HP	SERPINA1	9913.ENSBTAP00000071329	9913.ENSBTAP00000065816	0.073	0	0.837	0.842
HPX	TF	9913.ENSBTAP00000004635	9913.ENSBTAP00000009564	0.067	0	0.697	0.705
HPX	CP	9913.ENSBTAP00000004635	9913.ENSBTAP00000016137	0.077	0	0.709	0.72
HPX	SERPINA1	9913.ENSBTAP00000004635	9913.ENSBTAP00000065816	0.077	0	0.709	0.72
HPX	ALB	9913.ENSBTAP00000004635	9913.ENSBTAP00000022763	0.102	0.5	0.699	0.853
HPX	HP	9913.ENSBTAP00000004635	9913.ENSBTAP00000071329	0.073	0	0.918	0.921
HSPA5	PDIA6	9913.ENSBTAP00000052422	9913.ENSBTAP00000002508	0.328	0	0.842	0.889
HSPA5	A2M	9913.ENSBTAP00000052422	9913.ENSBTAP00000006167	0.083	0	0.808	0.817

HSPA5	OS9	9913.ENSBTAP00000052422	9913.ENSBTAP00000009410	0.496	0	0.635	0.808
HSPA5	PPIB	9913.ENSBTAP00000052422	9913.ENSBTAP00000022378	0.269	0.36	0.626	0.809
HSPA5	ALB	9913.ENSBTAP00000052422	9913.ENSBTAP00000022763	0	0	0.741	0.741
HSPA5	PDIA3	9913.ENSBTAP00000052422	9913.ENSBTAP00000022854	0.418	0.5	0.897	0.967
HSPA5	HSPA8	9913.ENSBTAP00000052422	9913.ENSBTAP00000058201	0.272	0.5	0.432	0.775
HSPA8	HSPA5	9913.ENSBTAP00000058201	9913.ENSBTAP00000052422	0.272	0.5	0.432	0.775
KNG1	ALB	9913.ENSBTAP00000048995	9913.ENSBTAP00000022763	0	0	0.835	0.835
KNG1	C3	9913.ENSBTAP00000048995	9913.ENSBTAP00000022979	0	0.4	0.585	0.74
KNG1	SERPINA1	9913.ENSBTAP00000048995	9913.ENSBTAP00000065816	0	0	0.729	0.729
KNG1	FGB	9913.ENSBTAP00000048995	9913.ENSBTAP00000067912	0.09	0	0.741	0.754
LALBA	LTF	9913.ENSBTAP00000060771	9913.ENSBTAP00000001704	0.052	0	0.998	0.998
LALBA	CSN1S1	9913.ENSBTAP00000060771	9913.ENSBTAP00000010119	0	0	0.936	0.936
LALBA	ALB	9913.ENSBTAP00000060771	9913.ENSBTAP00000022763	0	0	0.997	0.997
LALBA	CSN3	9913.ENSBTAP00000060771	9913.ENSBTAP00000028685	0	0	0.951	0.951
LALBA	LPO	9913.ENSBTAP00000060771	9913.ENSBTAP00000054415	0	0	0.969	0.969
LALBA	PAEP	9913.ENSBTAP00000060771	9913.ENSBTAP00000062602	0	0	0.999	0.999
LBP	CD14	9913.ENSBTAP00000022428	9913.ENSBTAP00000020009	0.187	0.6	0.843	0.944
LBP	HP	9913.ENSBTAP00000022428	9913.ENSBTAP00000071329	0	0	0.784	0.784
LCN2	LTF	9913.ENSBTAP00000018806	9913.ENSBTAP00000001704	0.064	0	0.817	0.822
LPL	APOH	9913.ENSBTAP00000017086	9913.ENSBTAP00000002492	0.043	0.9	0.29	0.926
LPL	CD36	9913.ENSBTAP00000017086	9913.ENSBTAP00000066341	0	0	0.758	0.758
LPO	LTF	9913.ENSBTAP00000054415	9913.ENSBTAP00000001704	0.063	0	0.966	0.966
LPO	ALB	9913.ENSBTAP00000054415	9913.ENSBTAP00000022763	0	0	0.796	0.796
LPO	CSN3	9913.ENSBTAP00000054415	9913.ENSBTAP00000028685	0	0	0.724	0.724
LPO	PAEP	9913.ENSBTAP00000054415	9913.ENSBTAP00000062602	0	0	0.903	0.903
LPO	LALBA	9913.ENSBTAP00000054415	9913.ENSBTAP00000060771	0	0	0.969	0.969

LTF	CSN1S1	9913.ENSBTAP00000001704	9913.ENSBTAP00000010119	0	0	0.777	0.777
LTF	HP	9913.ENSBTAP00000001704	9913.ENSBTAP00000071329	0.064	0	0.788	0.793
LTF	PGLYRP1	9913.ENSBTAP00000001704	9913.ENSBTAP00000003414	0	0	0.756	0.756
LTF	LCN2	9913.ENSBTAP00000001704	9913.ENSBTAP00000018806	0.064	0	0.817	0.822
LTF	CSN3	9913.ENSBTAP00000001704	9913.ENSBTAP00000028685	0	0	0.825	0.825
LTF	CLU	9913.ENSBTAP00000001704	9913.ENSBTAP00000007324	0.075	0.5	0.708	0.853
LTF	LPO	9913.ENSBTAP00000001704	9913.ENSBTAP00000054415	0.063	0	0.966	0.966
LTF	ALB	9913.ENSBTAP00000001704	9913.ENSBTAP00000022763	0	0	0.995	0.995
LTF	PAEP	9913.ENSBTAP00000001704	9913.ENSBTAP00000062602	0.045	0	0.998	0.998
LTF	LALBA	9913.ENSBTAP00000001704	9913.ENSBTAP00000060771	0.052	0	0.998	0.998
OS9	HSPA5	9913.ENSBTAP00000009410	9913.ENSBTAP00000052422	0.496	0	0.635	0.808
OS9	SERPINA1	9913.ENSBTAP00000009410	9913.ENSBTAP00000065816	0.501	0	0.648	0.817
PAEP	LTF	9913.ENSBTAP00000062602	9913.ENSBTAP00000001704	0.045	0	0.998	0.998
PAEP	CSN1S1	9913.ENSBTAP00000062602	9913.ENSBTAP00000010119	0	0	0.889	0.889
PAEP	ALB	9913.ENSBTAP00000062602	9913.ENSBTAP00000022763	0	0	0.998	0.998
PAEP	CSN3	9913.ENSBTAP00000062602	9913.ENSBTAP00000028685	0.045	0	0.997	0.997
PAEP	LPO	9913.ENSBTAP00000062602	9913.ENSBTAP00000054415	0	0	0.903	0.903
PAEP	LALBA	9913.ENSBTAP00000062602	9913.ENSBTAP00000060771	0	0	0.999	0.999
PCBP2	HNRNPA1	9913.ENSBTAP00000065293	9913.ENSBTAP00000013110	0.134	0.4	0.786	0.879
PCBP2	HNRNPA3	9913.ENSBTAP00000065293	9913.ENSBTAP00000028757	0.134	0.4	0.493	0.713
PDIA3	PDIA6	9913.ENSBTAP00000022854	9913.ENSBTAP00000002508	0.312	0	0.585	0.702
PDIA3	B2M	9913.ENSBTAP00000022854	9913.ENSBTAP00000016359	0.276	0.5	0.872	0.949
PDIA3	HSPA5	9913.ENSBTAP00000022854	9913.ENSBTAP00000052422	0.418	0.5	0.897	0.967
PDIA6	PDIA3	9913.ENSBTAP00000002508	9913.ENSBTAP00000022854	0.312	0	0.585	0.702
PDIA6	HSPA5	9913.ENSBTAP00000002508	9913.ENSBTAP00000052422	0.328	0	0.842	0.889
PGLYRP1	LTF	9913.ENSBTAP00000003414	9913.ENSBTAP00000001704	0	0	0.756	0.756

PLG	CSN3	9913.ENSBTAP00000001674	9913.ENSBTAP00000028685	0.048	0	0.722	0.724
PLG	A2M	9913.ENSBTAP00000001674	9913.ENSBTAP00000006167	0.141	0	0.687	0.719
PLG	CLEC3B	9913.ENSBTAP00000001674	9913.ENSBTAP00000024391	0.063	0	0.835	0.839
PLG	SERPINC1	9913.ENSBTAP00000001674	9913.ENSBTAP00000072452	0.104	0	0.779	0.794
PLG	C3	9913.ENSBTAP00000001674	9913.ENSBTAP00000022979	0.141	0.9	0.711	0.973
PLG	ALB	9913.ENSBTAP00000001674	9913.ENSBTAP00000022763	0.047	0	0.855	0.855
PLIN2	CD36	9913.ENSBTAP00000007519	9913.ENSBTAP00000066341	0.112	0	0.697	0.719
PLIN2	BTN1A1	9913.ENSBTAP00000007519	9913.ENSBTAP00000042450	0.063	0	0.735	0.741
PLIN2	XDH	9913.ENSBTAP00000007519	9913.ENSBTAP00000061483	0	0	0.772	0.772
PPIB	HSPA5	9913.ENSBTAP00000022378	9913.ENSBTAP00000052422	0.269	0.36	0.626	0.809
RBP4	GC	9913.ENSBTAP00000057660	9913.ENSBTAP00000062210	0	0	0.747	0.747
SERPINA1	HPX	9913.ENSBTAP00000065816	9913.ENSBTAP00000004635	0.077	0	0.709	0.72
SERPINA1	A2M	9913.ENSBTAP00000065816	9913.ENSBTAP00000006167	0	0	0.862	0.862
SERPINA1	OS9	9913.ENSBTAP00000065816	9913.ENSBTAP00000009410	0.501	0	0.648	0.817
SERPINA1	TF	9913.ENSBTAP00000065816	9913.ENSBTAP00000009564	0.067	0	0.693	0.701
SERPINA1	CFB	9913.ENSBTAP00000065816	9913.ENSBTAP00000009800	0.071	0	0.707	0.716
SERPINA1	CP	9913.ENSBTAP00000065816	9913.ENSBTAP00000016137	0.077	0	0.842	0.847
SERPINA1	ALB	9913.ENSBTAP00000065816	9913.ENSBTAP00000022763	0.063	0	0.883	0.885
SERPINA1	C3	9913.ENSBTAP00000065816	9913.ENSBTAP00000022979	0	0	0.799	0.799
SERPINA1	KNG1	9913.ENSBTAP00000065816	9913.ENSBTAP00000048995	0	0	0.729	0.729
SERPINA1	GC	9913.ENSBTAP00000065816	9913.ENSBTAP00000062210	0.077	0	0.689	0.701
SERPINA1	HP	9913.ENSBTAP00000065816	9913.ENSBTAP00000071329	0.073	0	0.837	0.842
SERPINC1	PLG	9913.ENSBTAP00000072452	9913.ENSBTAP00000001674	0.104	0	0.779	0.794
SERPINC1	FGA	9913.ENSBTAP00000072452	9913.ENSBTAP00000002145	0.043	0	0.707	0.708
TF	HPX	9913.ENSBTAP00000009564	9913.ENSBTAP00000004635	0.067	0	0.697	0.705
TF	A2M	9913.ENSBTAP00000009564	9913.ENSBTAP00000006167	0	0	0.795	0.795

TF	HP	9913.ENSBTAP00000009564	9913.ENSBTAP00000071329	0.064	0	0.744	0.75
TF	SERPINA1	9913.ENSBTAP00000009564	9913.ENSBTAP00000065816	0.067	0	0.693	0.701
TF	C3	9913.ENSBTAP00000009564	9913.ENSBTAP00000022979	0.044	0	0.748	0.748
TF	CP	9913.ENSBTAP00000009564	9913.ENSBTAP00000016137	0.078	0	0.77	0.778
TF	GC	9913.ENSBTAP00000009564	9913.ENSBTAP00000062210	0.067	0	0.799	0.804
TF	ALB	9913.ENSBTAP00000009564	9913.ENSBTAP00000022763	0	0	0.835	0.835
TKT	ALDOA	9913.ENSBTAP00000004892	9913.ENSBTAP00000057869	0.077	0.939	0.631	0.977
TPM1	VIM	9913.ENSBTAP00000063733	9913.ENSBTAP00000024572	0	0.5	0.529	0.754
VIM	DES	9913.ENSBTAP00000024572	9913.ENSBTAP00000007041	0	0.8	0	0.8
VIM	TPM1	9913.ENSBTAP00000024572	9913.ENSBTAP00000063733	0	0.5	0.529	0.754
VIM	CDH1	9913.ENSBTAP00000024572	9913.ENSBTAP00000073179	0.043	0	0.865	0.865
XDH	PLIN2	9913.ENSBTAP00000061483	9913.ENSBTAP00000007519	0	0	0.772	0.772
XDH	BTN1A1	9913.ENSBTAP00000061483	9913.ENSBTAP00000042450	0	0.9	0.861	0.985

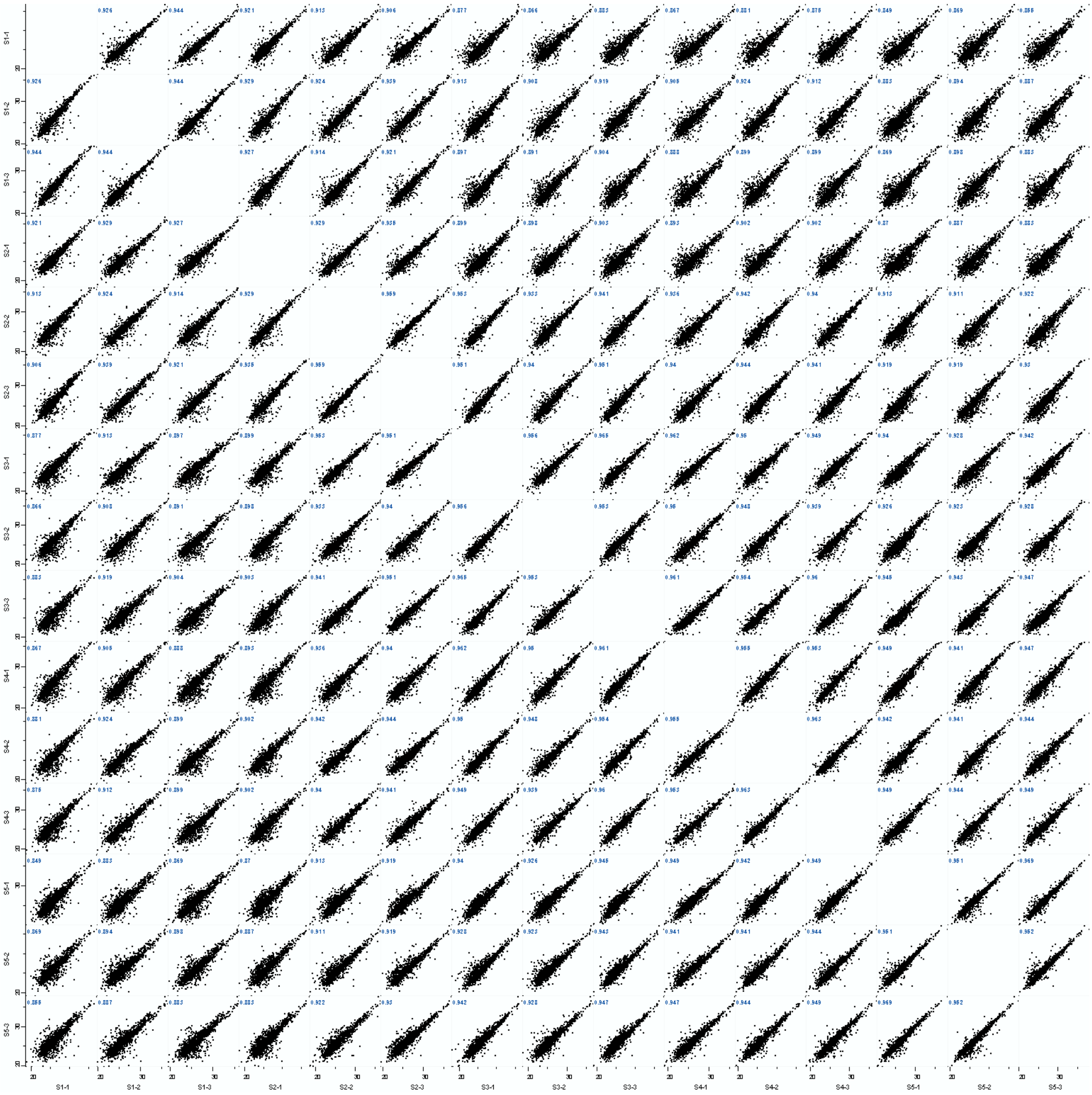

Figure S1. Pearson correlation matrix for the quantified whey proteins from three replicates for each group among the studied milk groups.

tr|G3MYN2|G3MYN2_BOVIN LWEGSTR

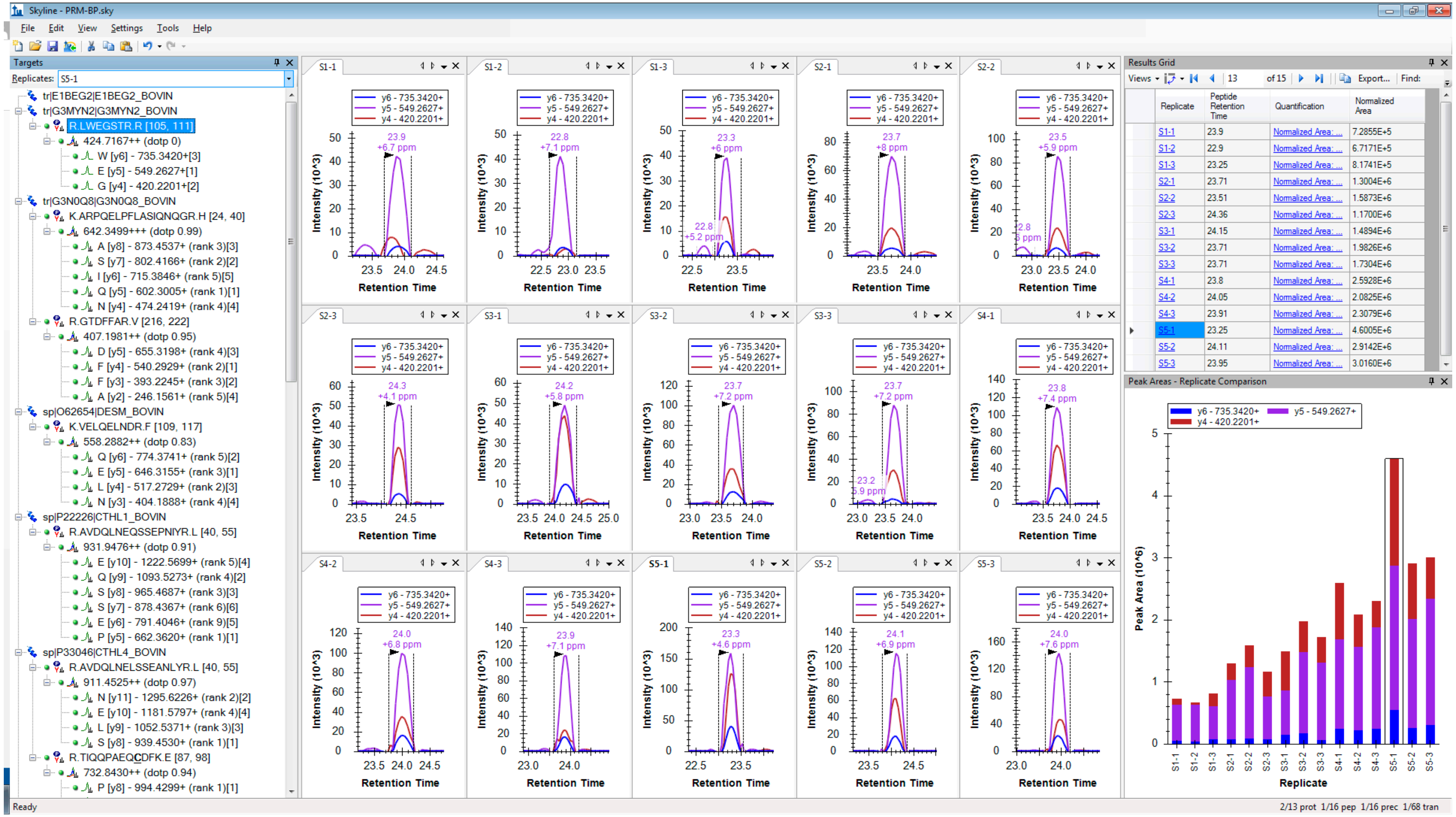

tr|G3N0Q8|G3N0Q8_BOVIN ARPQELPFLASIQNQGR

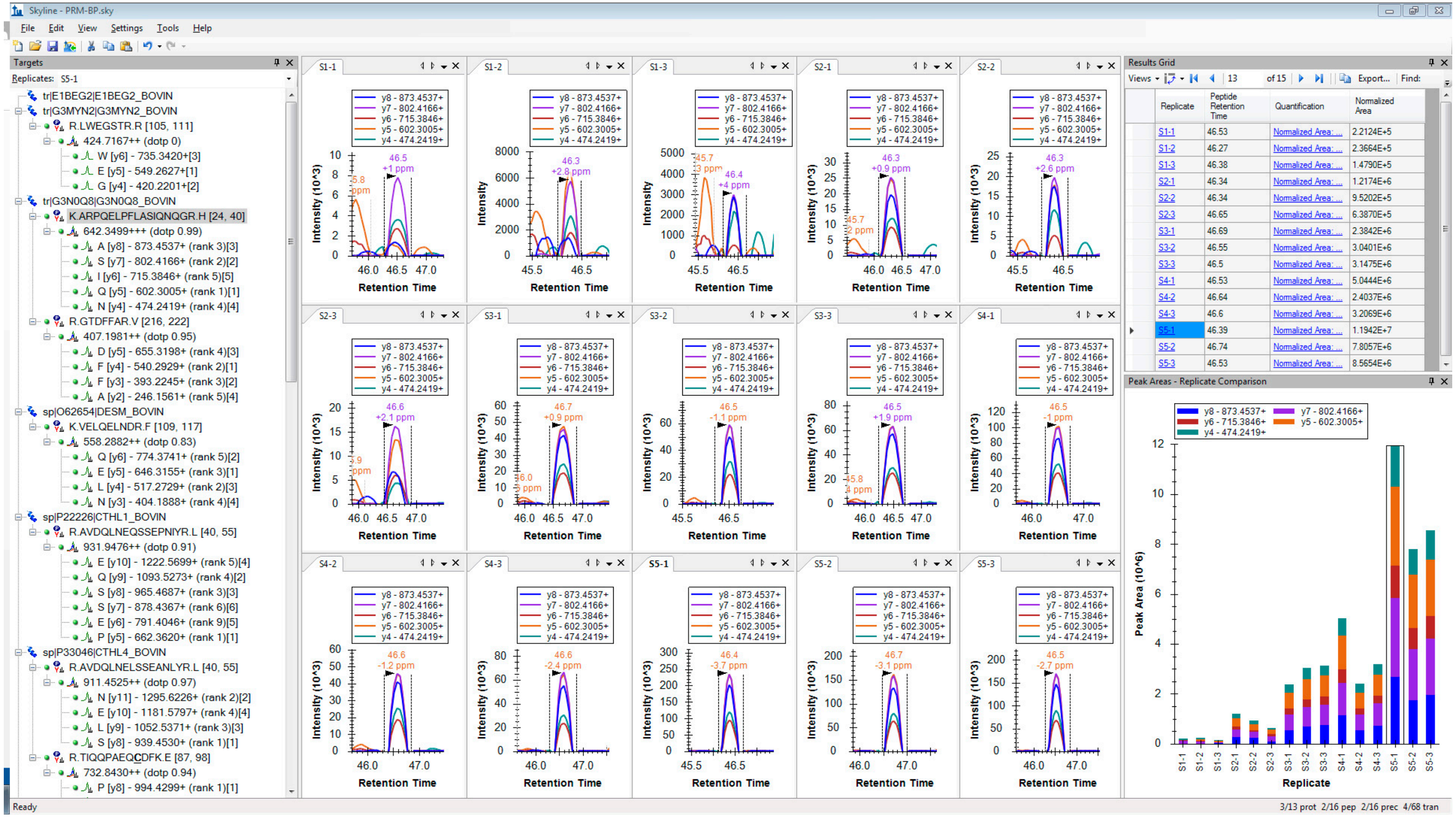

tr|G3N0Q8|G3N0Q8_BOVIN GTDFFAR

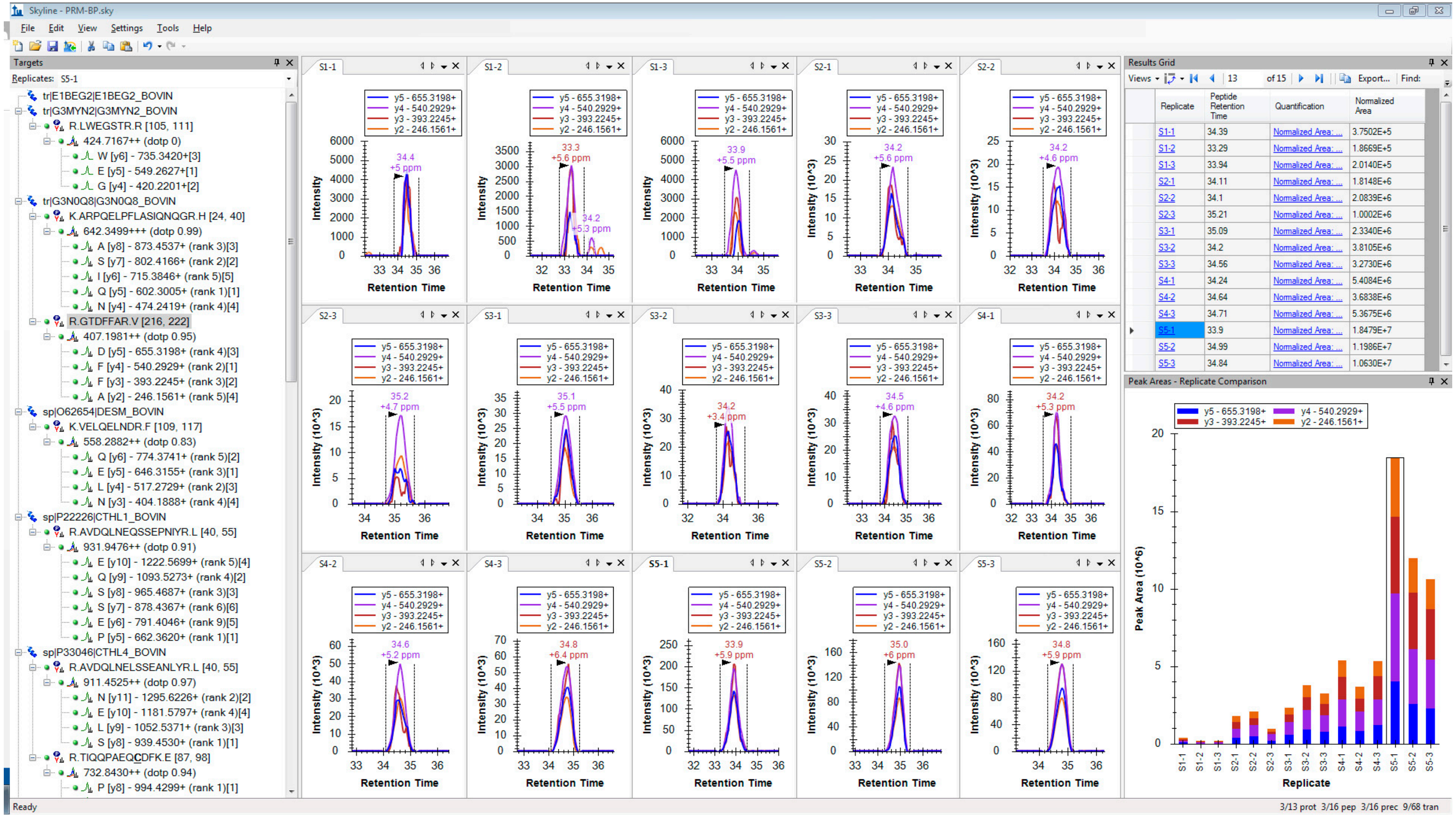

sp|O62654|DESM_BOVIN VELQELNDR

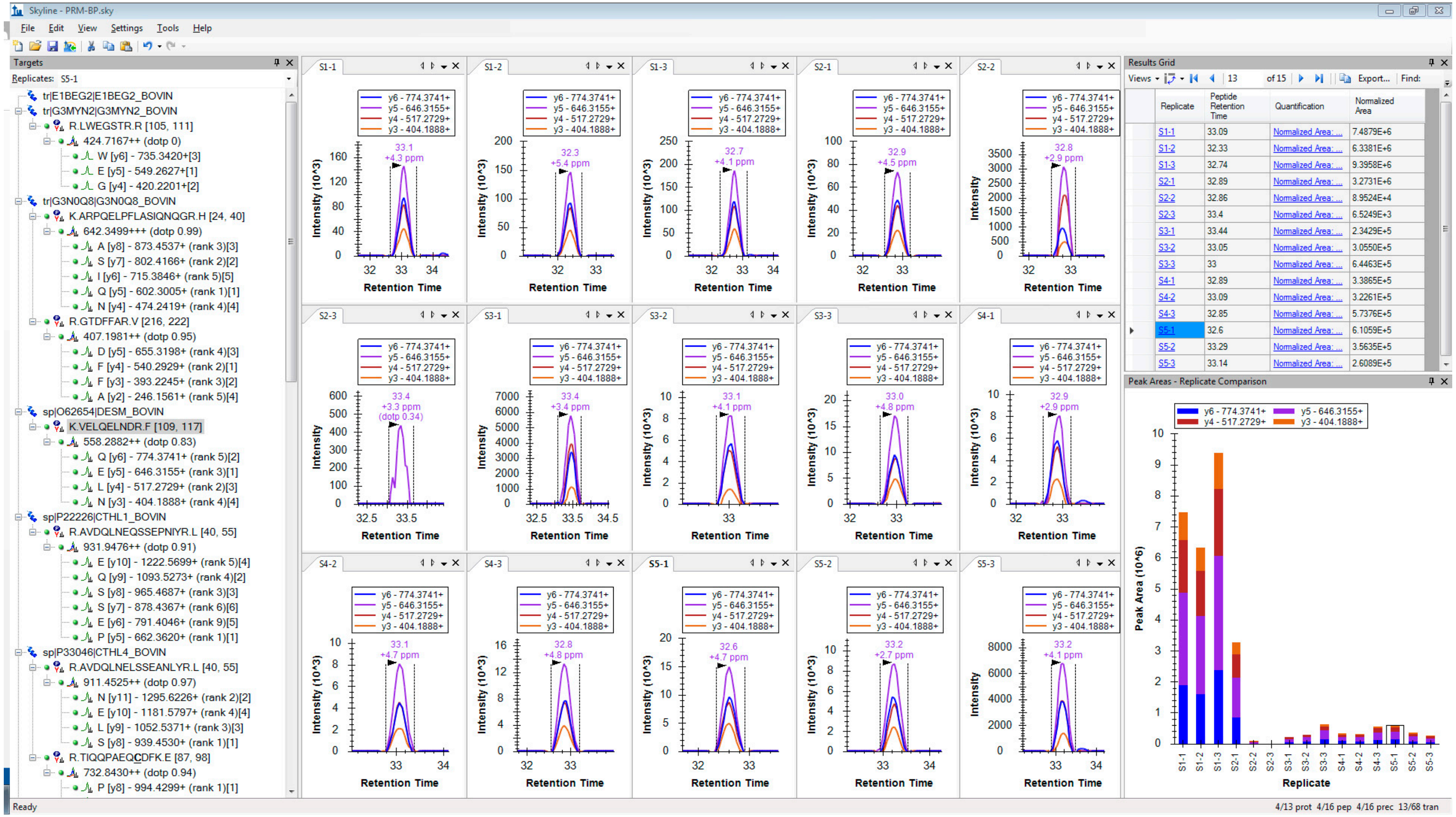

sp|P22226|CTHL1_BOVIN AVDQLNEQSSEPNIYR

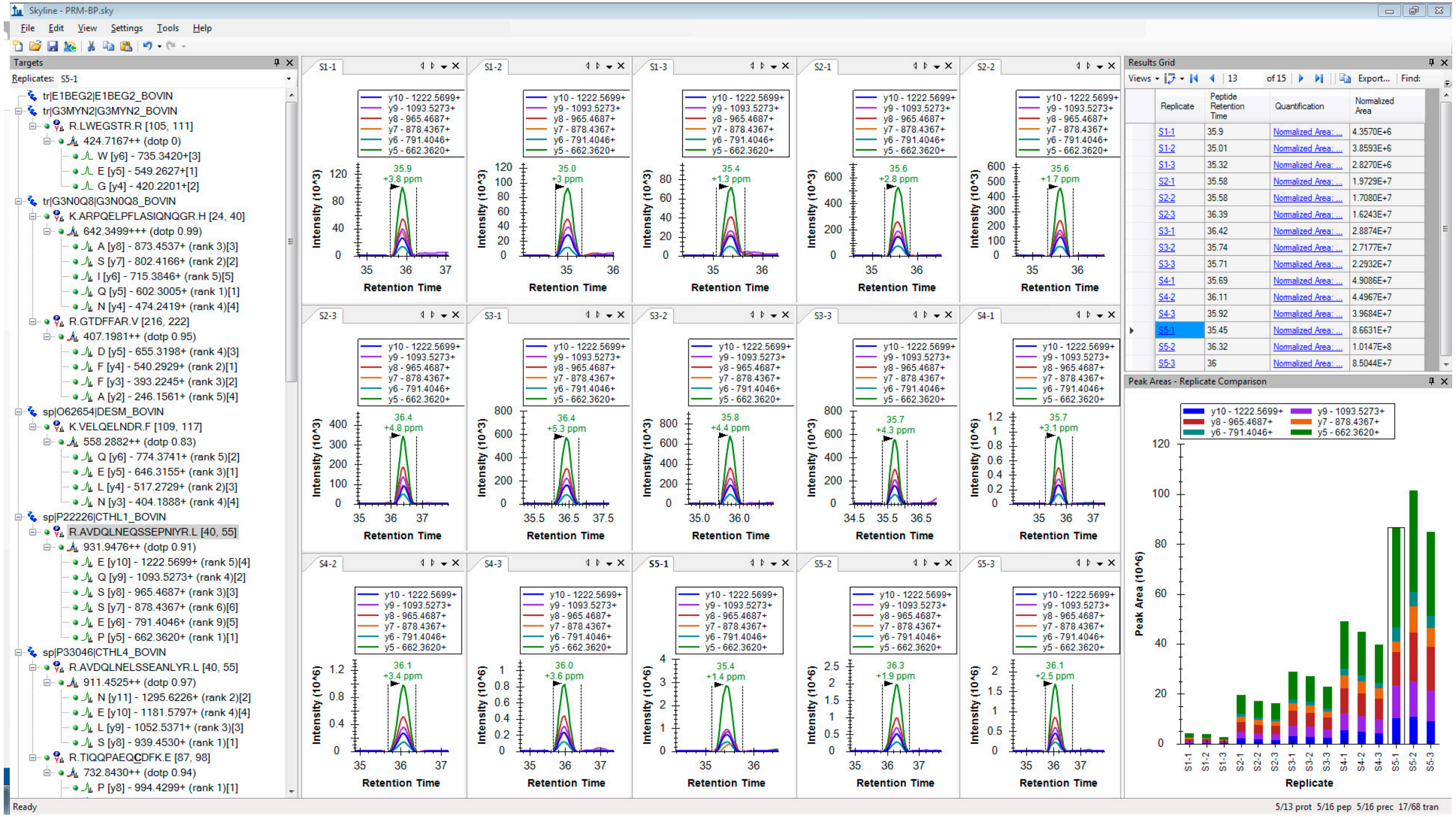

sp|P33046|CTHL4_BOVIN AVDQLNELSSEANLYR

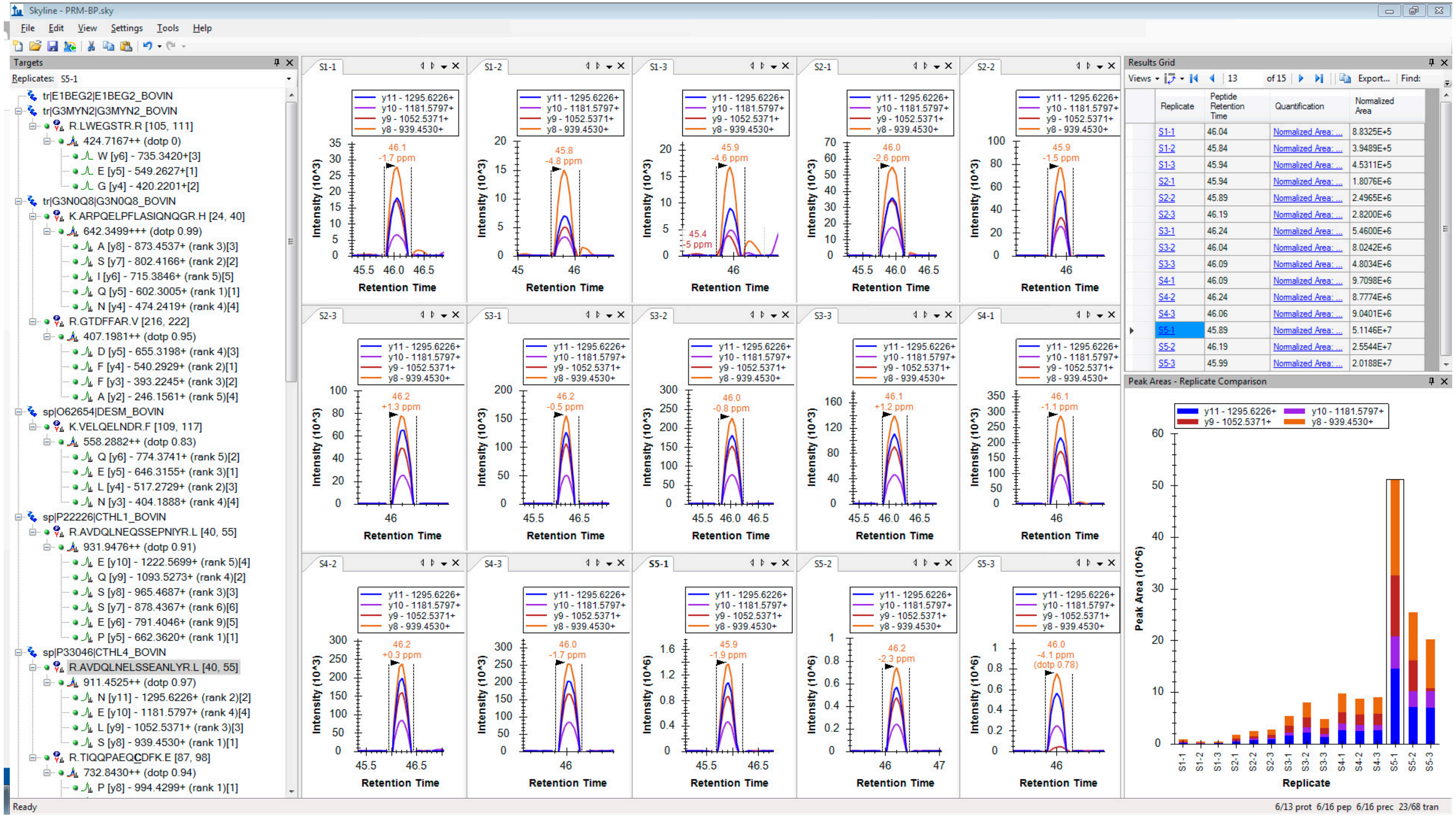

sp|P33046|CTHL4_BOVIN TIQQPAEQCDFK

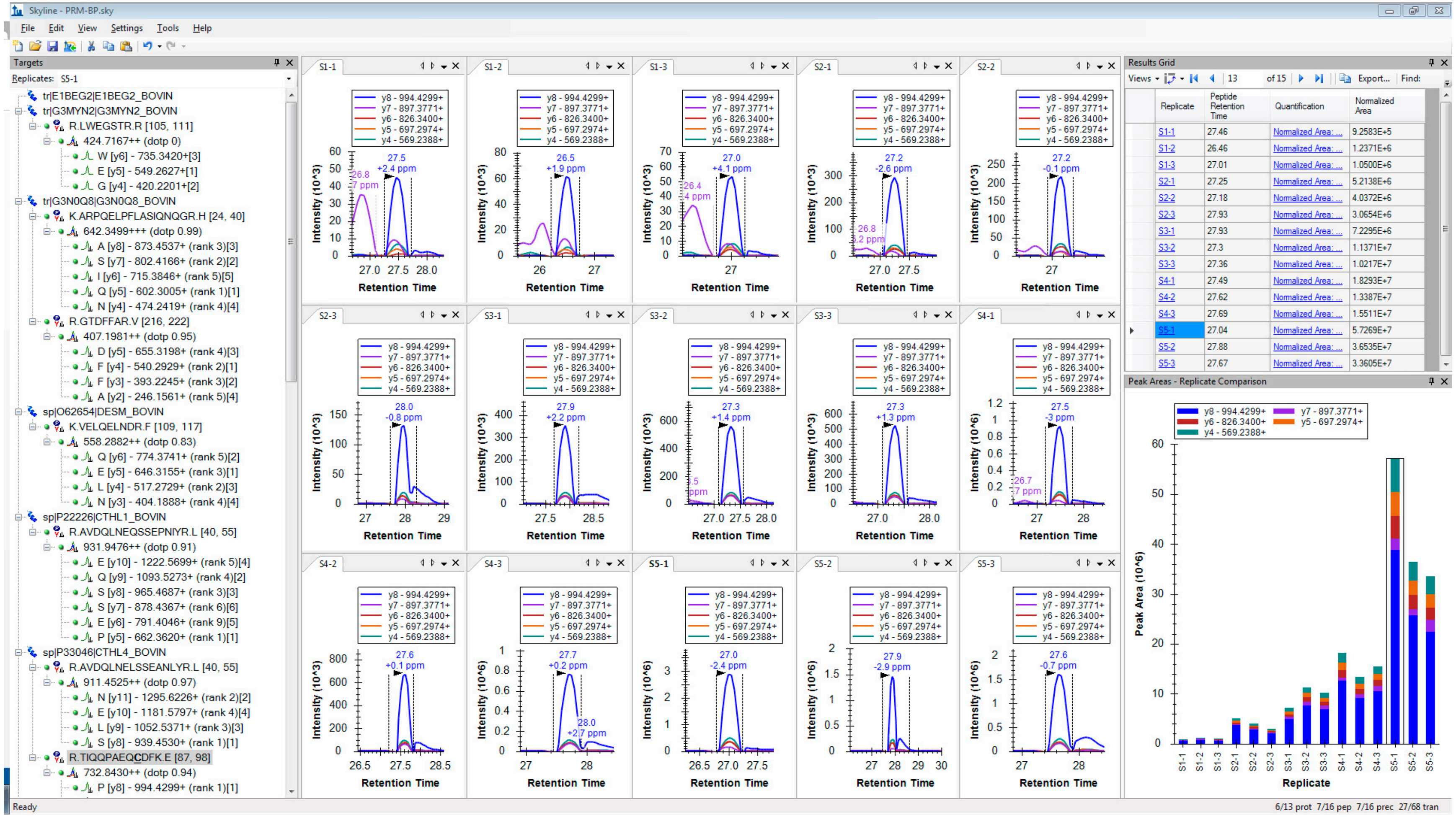

sp|P54228|CTHL6_BOVIN TSQQPAEQCDFK

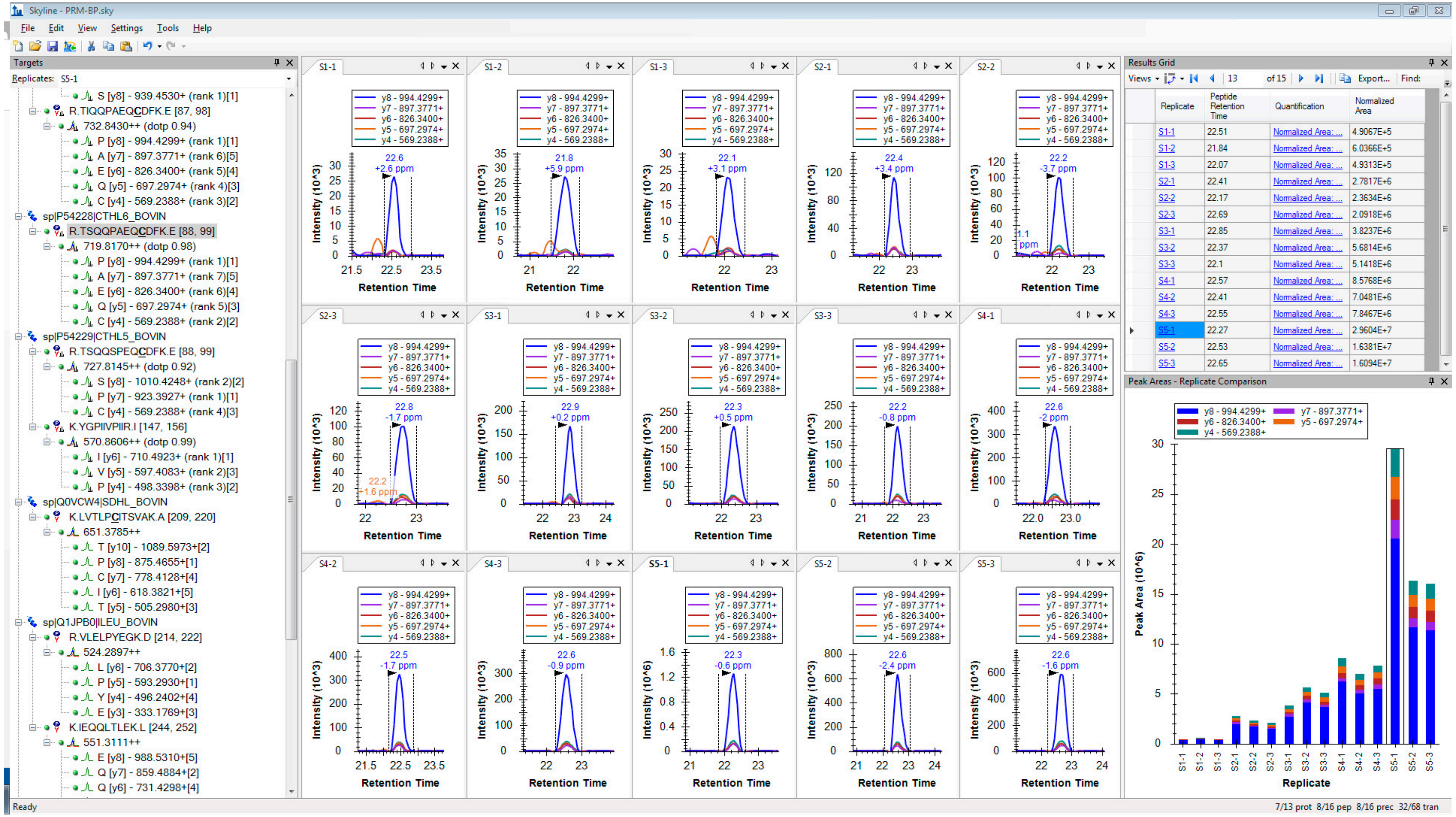

sp|P54229|CTHL5_BOVIN TSQQSPEQCDFK

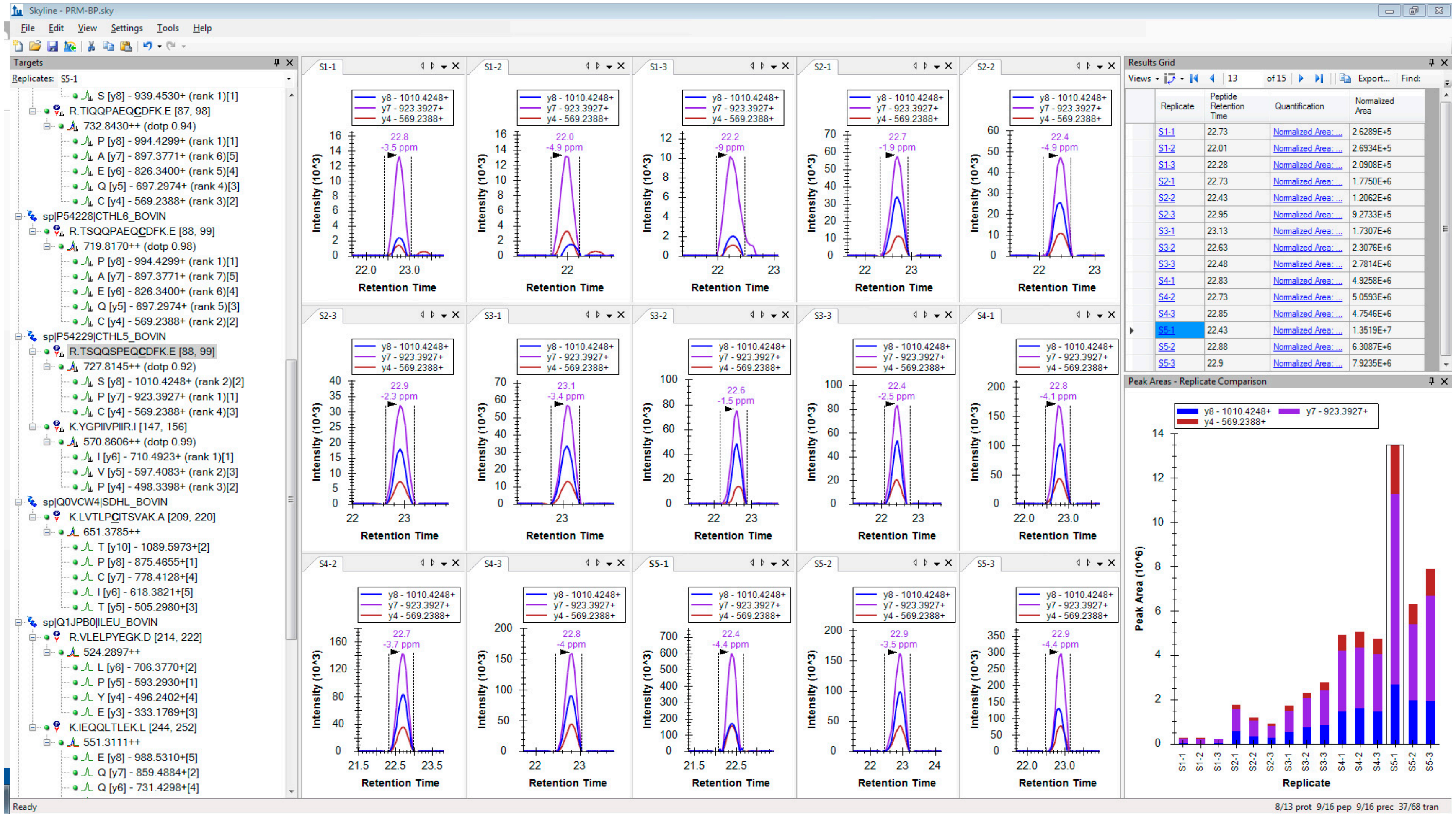

sp|P54229|CTHL5_BOVIN YGPIIVPIIR

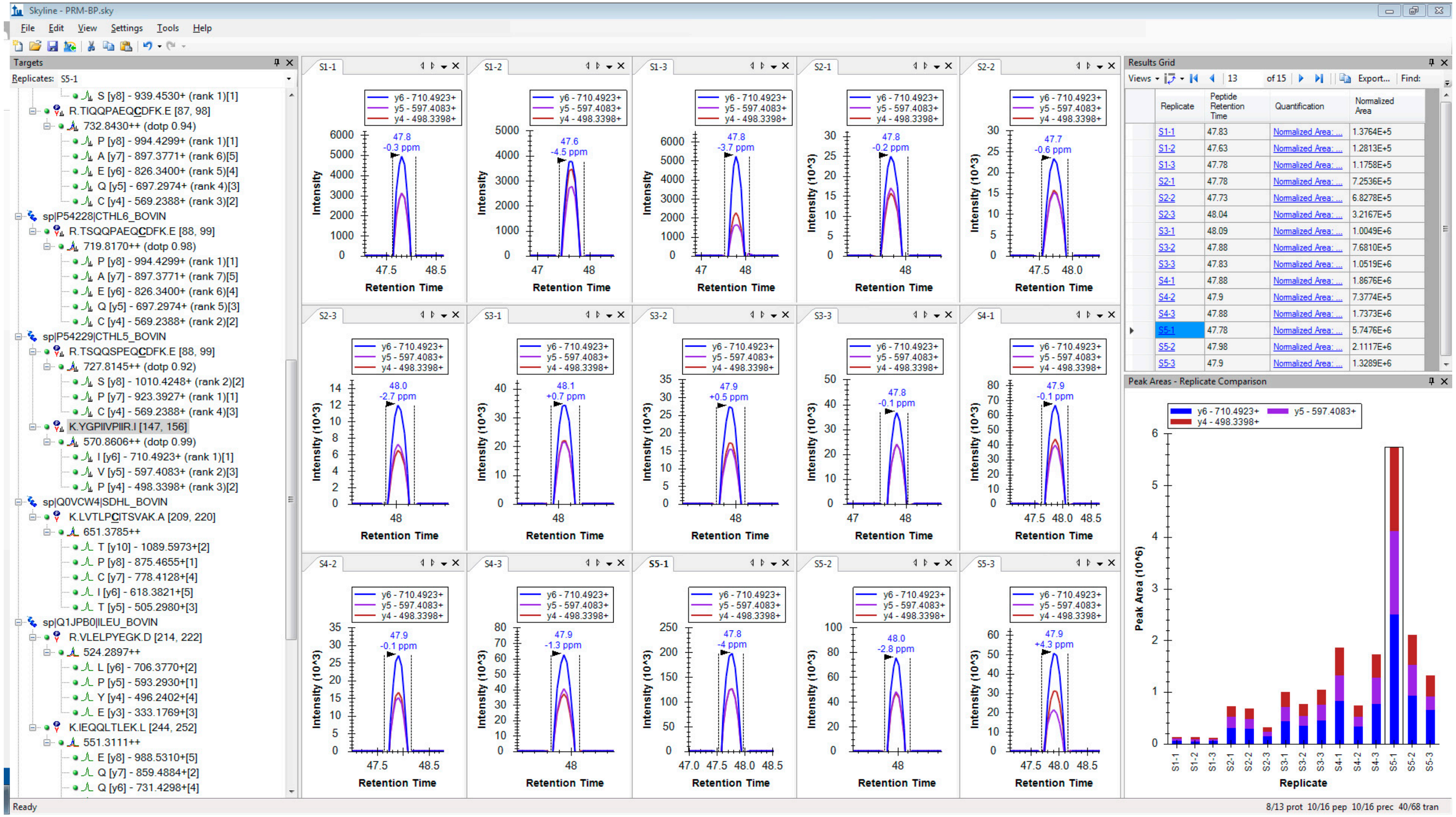

sp|Q0VCW4|SDHL_BOVIN LVTLPCITSVAK

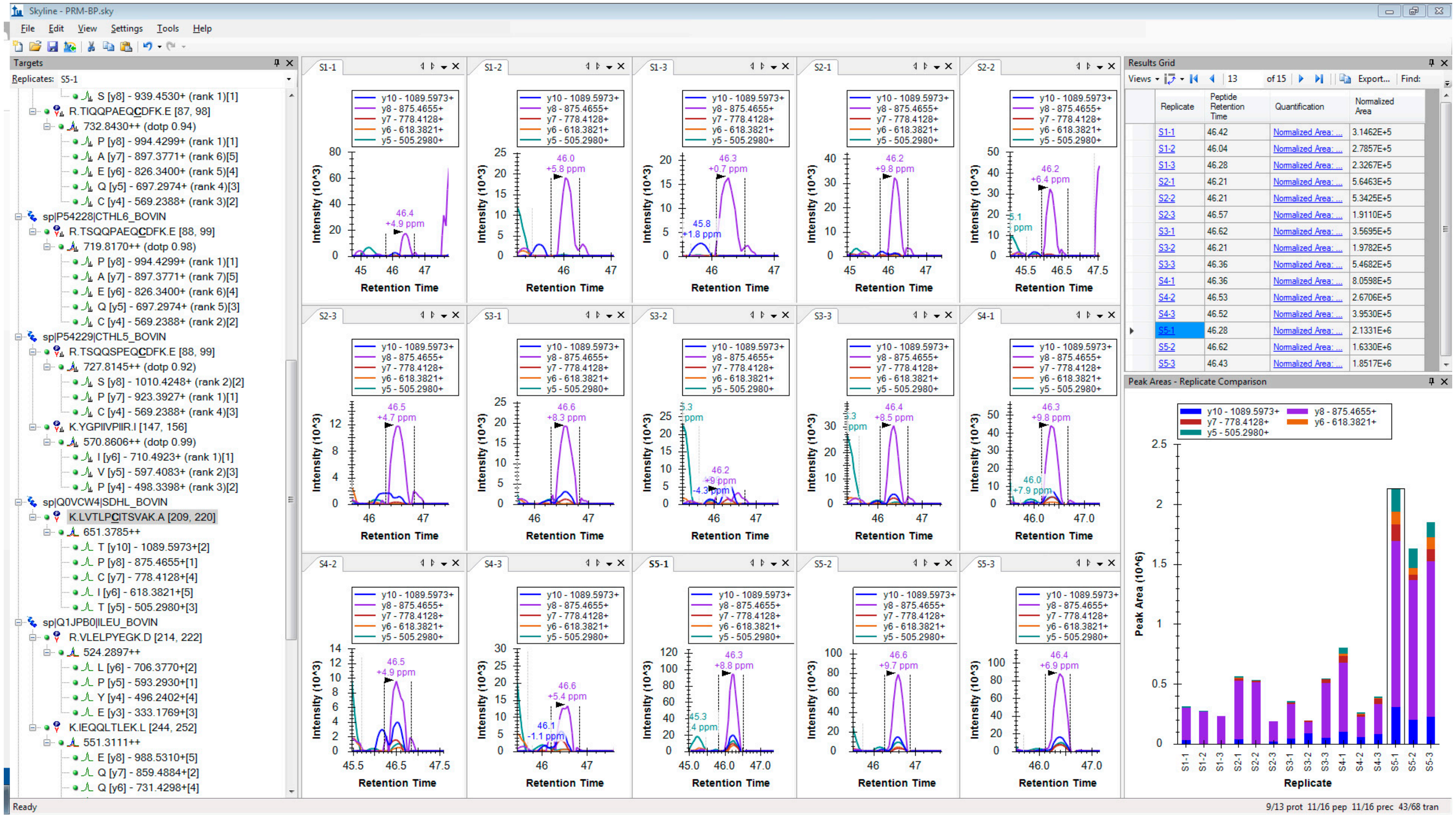

sp|Q1JPB0|ILEU_BOVIN VLELPYEGK

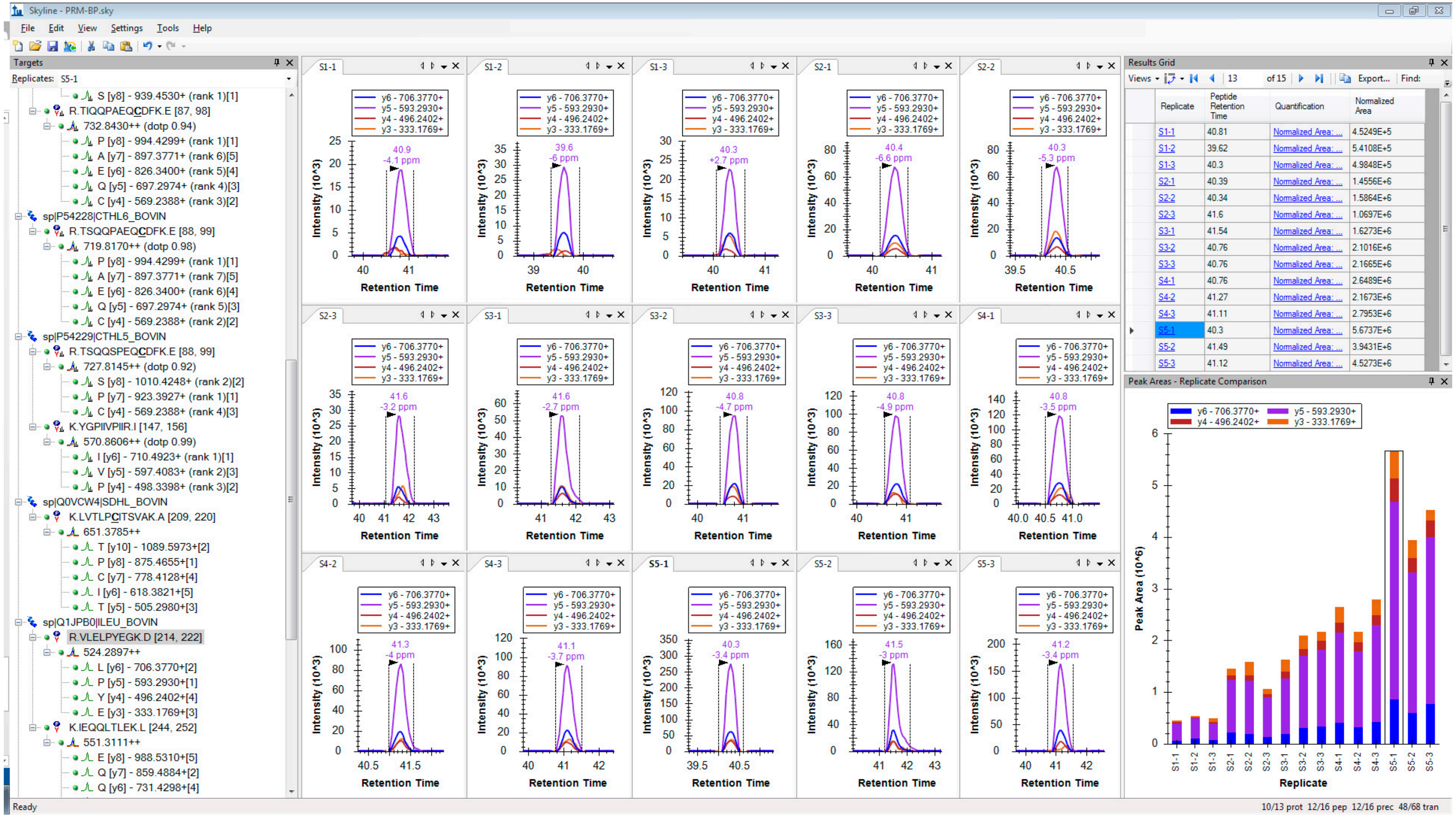

sp|Q1JPB0|ILEU_BOVIN IEQQLTLEK

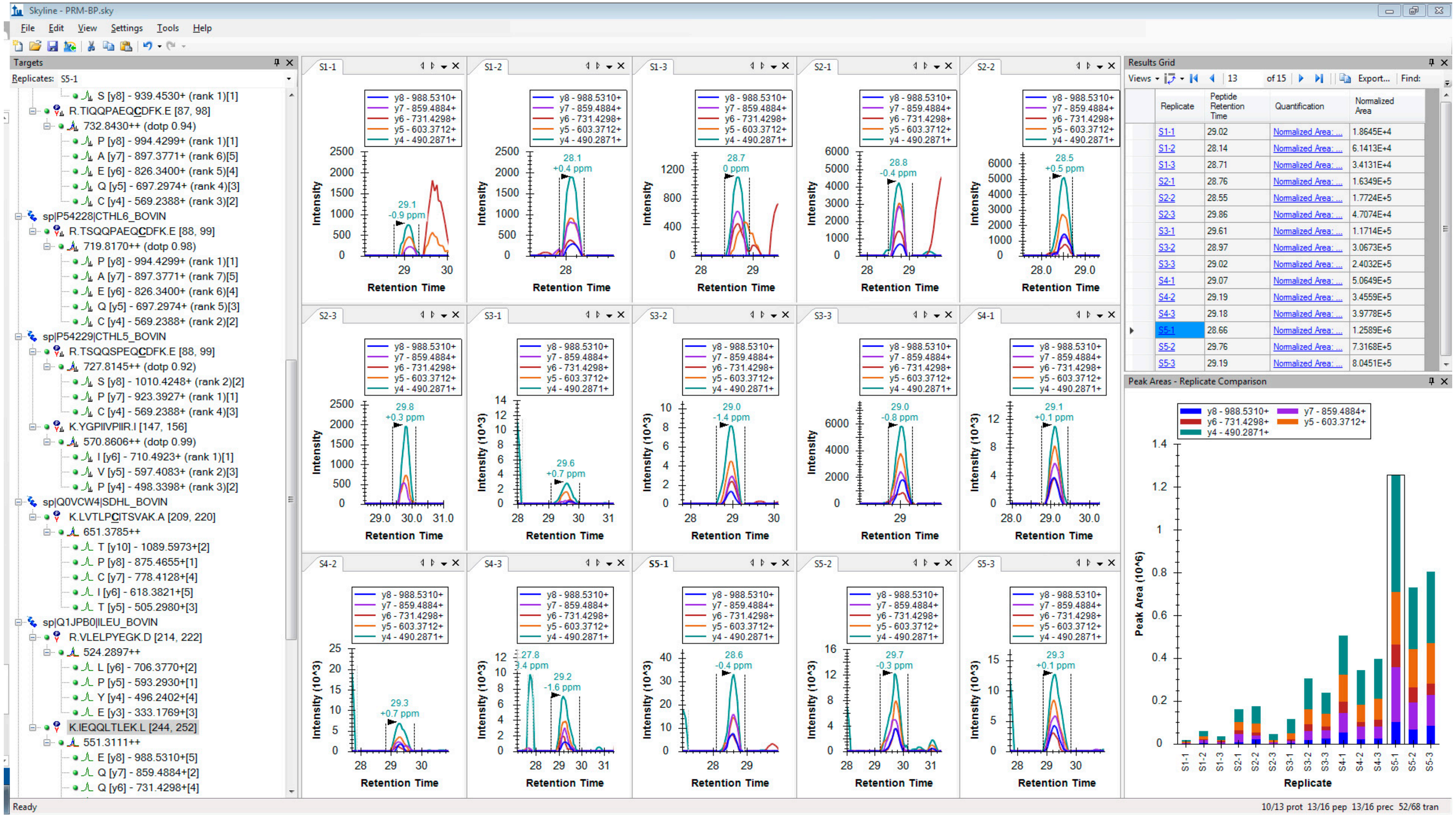

tr|Q3ZC00|Q3ZC00_BOVIN AYYHLLEQVAPK

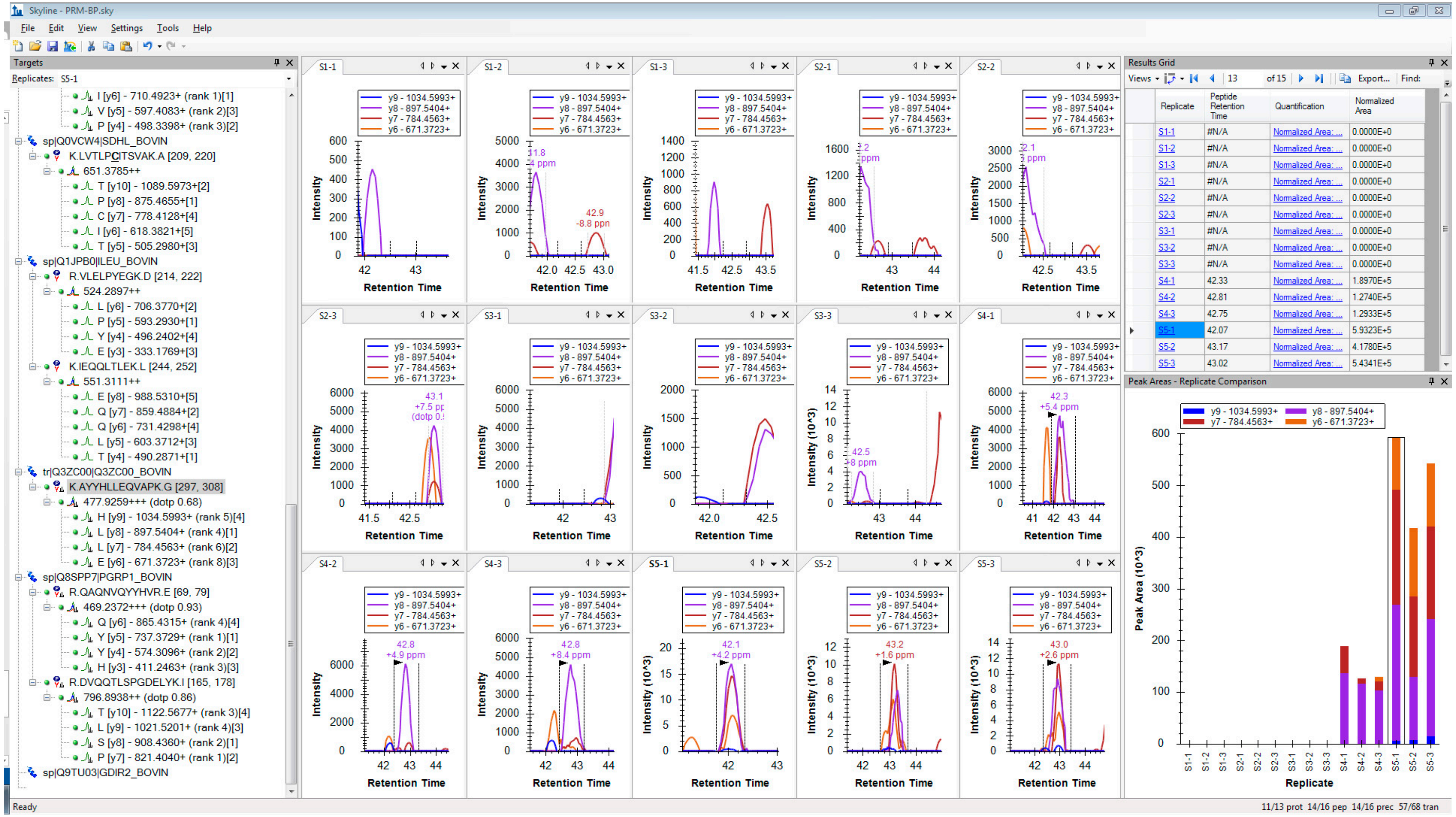

sp|Q8SPP7|PGRP1_BOVIN QAQNVQYYHVR

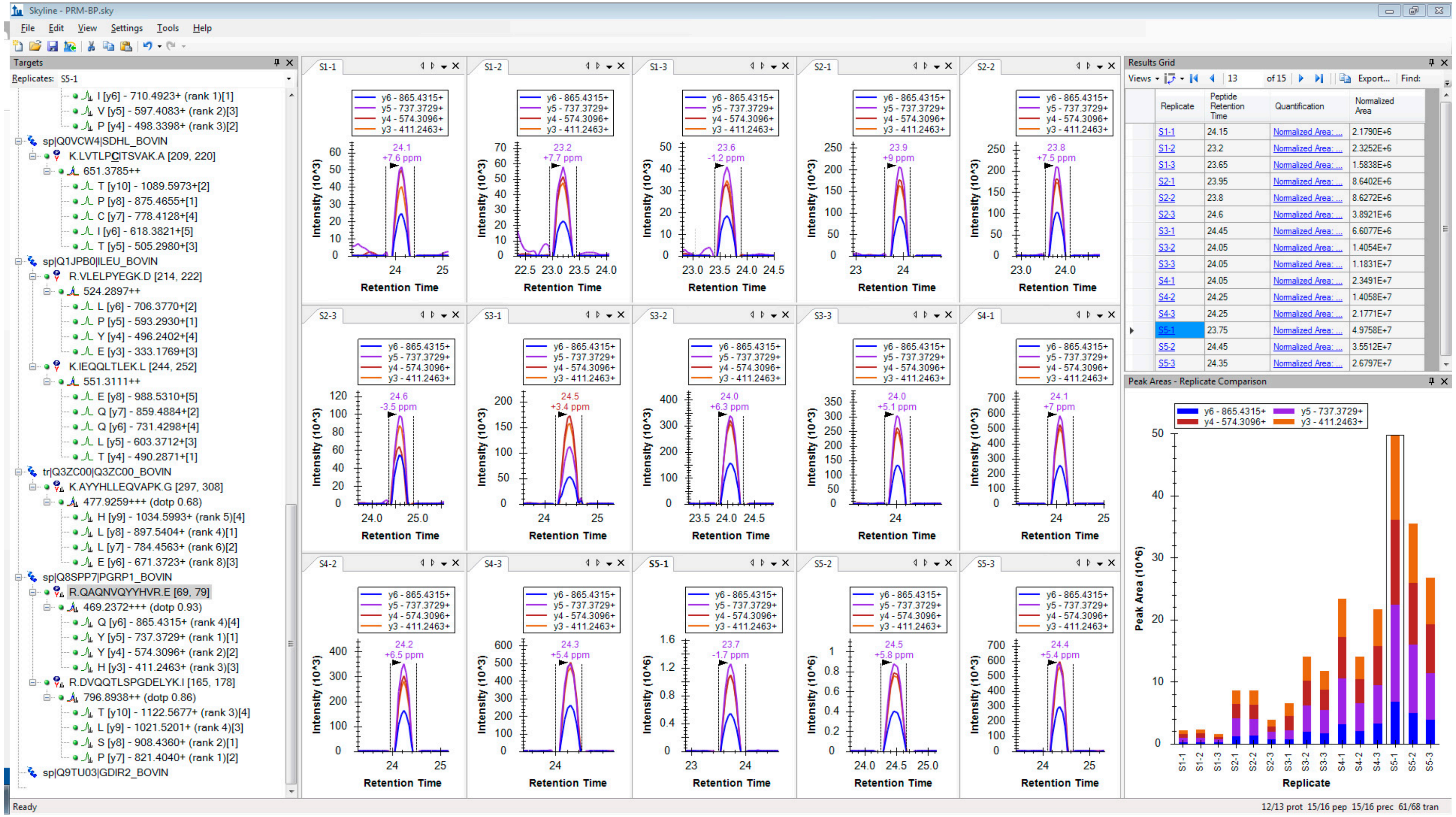

sp|Q8SPP7|PGRP1_BOVIN DVQQTLSPGDELYK

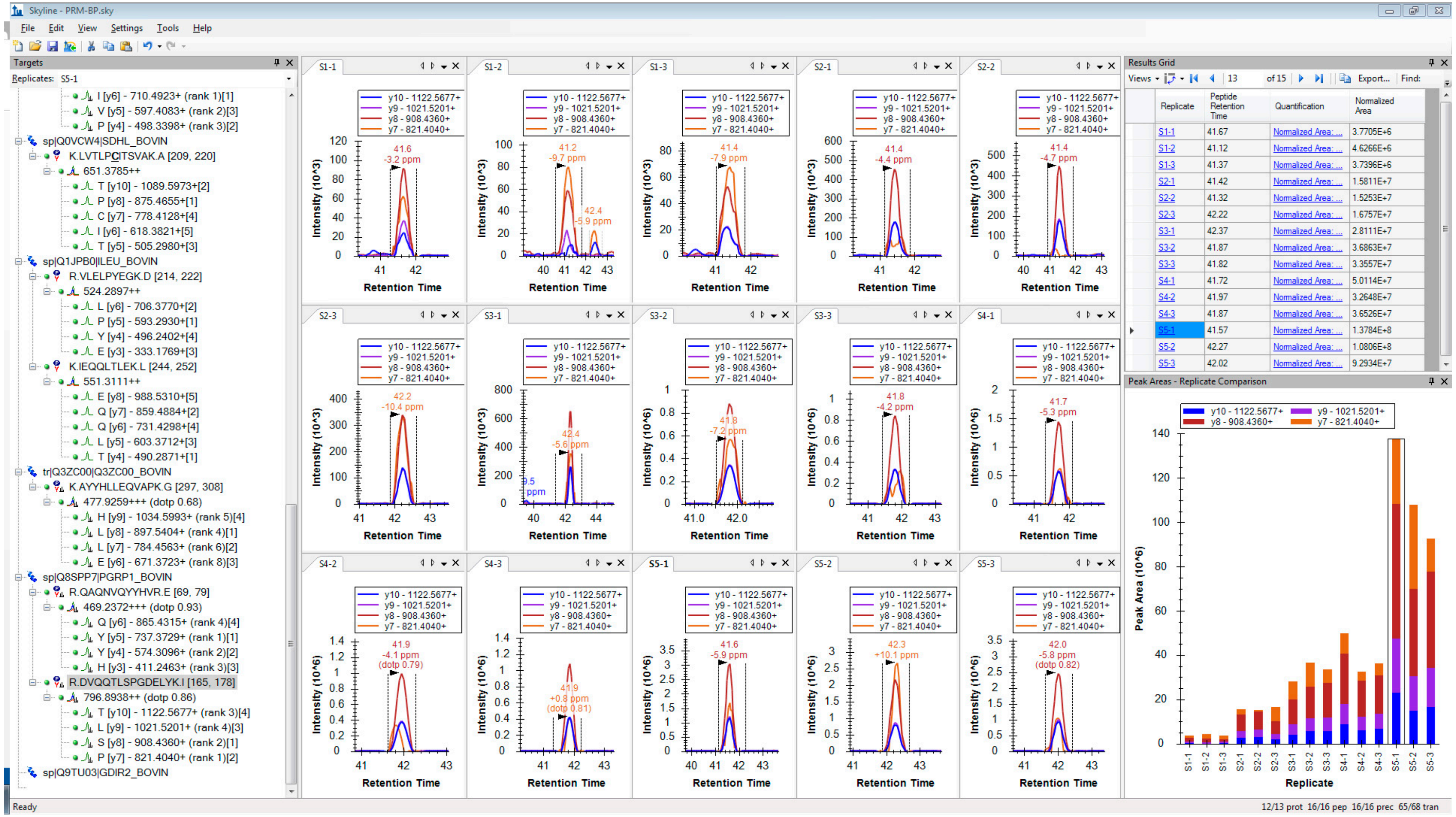

Figure S2. Results of target peptides from the different somatic cell count milk groups analyzed by Skyline software.

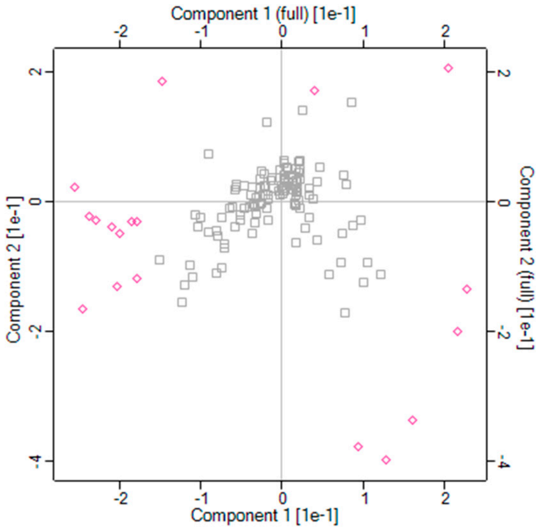

Figure S3. Loading plots of the protein components of milk whey with the different somatic cell counts.
